# Identification of Genomic Regions Associated with Phenotypic Variation between Dog Breeds using Selection Mapping

**DOI:** 10.1371/journal.pgen.1002316

**Published:** 2011-10-13

**Authors:** Amaury Vaysse, Abhirami Ratnakumar, Thomas Derrien, Erik Axelsson, Gerli Rosengren Pielberg, Snaevar Sigurdsson, Tove Fall, Eija H. Seppälä, Mark S. T. Hansen, Cindy T. Lawley, Elinor K. Karlsson, The LUPA Consortium, Danika Bannasch, Carles Vilà, Hannes Lohi, Francis Galibert, Merete Fredholm, Jens Häggström, Åke Hedhammar, Catherine André, Kerstin Lindblad-Toh, Christophe Hitte, Matthew T. Webster

**Affiliations:** 1 Institut de Génétique et Développement de Rennes, CNRS-UMR6061, Université de Rennes 1, Rennes, France; 2 Science for Life Laboratory, Department of Medical Biochemistry and Microbiology, Uppsala University, Uppsala, Sweden; 3 Broad Institute of Harvard and Massachusetts Institute of Technology, Cambridge, Massachusetts, United States of America; 4 Department of Medical Epidemiology and Biostatistics, Karolinska Institute, Stockholm, Sweden; 5 Department of Veterinary Biosciences, Research Programs Unit, Molecular Medicine, University of Helsinki and Folkhälsan Research Center, Helsinki, Finland; 6 Illumina, San Diego, California, United States of America; 7 FAS Center for Systems Biology, Harvard University, Cambridge, Massachusetts, United States of America; 8 Department of Population Health and Reproduction, School of Veterinary Medicine, University of California Davis, Davis, California, United States of America; 9 Department of Integrative Ecology, Doñana Biological Station (CSIC), Seville, Spain; 10 Faculty of Life Sciences, Division of Genetics and Bioinformatics, Department of Basic Animal and Veterinary Sciences, University of Copenhagen, Frederiksberg, Denmark; 11 Department of Clinical Sciences, Swedish University of Agricultural Sciences, Uppsala, Sweden; University of Washington, United States of America

## Abstract

The extraordinary phenotypic diversity of dog breeds has been sculpted by a unique population history accompanied by selection for novel and desirable traits. Here we perform a comprehensive analysis using multiple test statistics to identify regions under selection in 509 dogs from 46 diverse breeds using a newly developed high-density genotyping array consisting of >170,000 evenly spaced SNPs. We first identify 44 genomic regions exhibiting extreme differentiation across multiple breeds. Genetic variation in these regions correlates with variation in several phenotypic traits that vary between breeds, and we identify novel associations with both morphological and behavioral traits. We next scan the genome for signatures of selective sweeps in single breeds, characterized by long regions of reduced heterozygosity and fixation of extended haplotypes. These scans identify hundreds of regions, including 22 blocks of homozygosity longer than one megabase in certain breeds. Candidate selection loci are strongly enriched for developmental genes. We chose one highly differentiated region, associated with body size and ear morphology, and characterized it using high-throughput sequencing to provide a list of variants that may directly affect these traits. This study provides a catalogue of genomic regions showing extreme reduction in genetic variation or population differentiation in dogs, including many linked to phenotypic variation. The many blocks of reduced haplotype diversity observed across the genome in dog breeds are the result of both selection and genetic drift, but extended blocks of homozygosity on a megabase scale appear to be best explained by selection. Further elucidation of the variants under selection will help to uncover the genetic basis of complex traits and disease.

## Author Summary

There are hundreds of dog breeds that exhibit massive differences in appearance and behavior sculpted by tightly controlled selective breeding. This large-scale natural experiment has provided an ideal resource that geneticists can use to search for genetic variants that control these differences. With this goal, we developed a high-density array that surveys variable sites at more than 170,000 positions in the dog genome and used it to analyze genetic variation in 46 breeds. We identify 44 chromosomal regions that are extremely variable between breeds and are likely to control many of the traits that vary between them, including curly tails and sociality. Many other regions also bear the signature of strong artificial selection. We characterize one such region, known to associate with body size and ear type, in detail using “next-generation” sequencing technology to identify candidate mutations that may control these traits. Our results suggest that artificial selection has targeted genes involved in development and metabolism and that it may have increased the incidence of disease in dog breeds. Knowledge of these regions will be of great importance for uncovering the genetic basis of variation between dog breeds and for finding mutations that cause disease.

## Introduction

There are more than 400 breeds of domestic dog, which exhibit characteristic variation in morphology, physiology and behavior. This astonishing phenotypic diversity has been molded by two main phases of evolution: 1) the initial domestication from wolves more than 15,000 years ago, where dogs became adapted to life in closer proximity to humans and 2) the formation of distinct breeds in the last few hundred years, where humans chose small groups of dogs from the gene pool and strongly selected for novel and desirable traits [1], [2]. A by-product of these processes has been that many dog breeds suffer from a high incidence of inherited disorders [3], [4].

Its unique population history makes the dog an ideal model organism for mapping the genetic basis of phenotypic traits due to extensive linkage disequilibrium (LD) and a reduction in haplotype diversity due to genetic drift in isolated populations [3]-[5]. Another major advantage of the canine model is that much of the variation in morphological characteristics in dogs appears to be governed by a relatively small number of genetic variants with large effect [6]. This is likely because novel variants with large effects are preserved by artificial selection. This is in strong contrast to humans where morphological variation in traits such as height appears to be controlled by hundreds of loci with small effects, which have proven extremely difficult to catalogue [7]. Identifying the targets of artificial selection in dog breeds is therefore an extremely promising approach for identifying genetic variants involved in phenotypic variation, which could greatly facilitate the identification of similar variants and novel molecular pathways in humans.

Several loci have now been identified that control variation in morphological traits between dog breeds. In some cases, variation in a trait occurs within a breed, and long blocks of LD can be used to identify the locus responsible using genome wide association studies (GWAS). Using this approach loci involved in traits including size (IGF1) [8], coat type (RSPO2, FGF5, KRT71) [9] and coat color (MITF, CBD103) [10], [11] were identified in single breeds, and it was shown that variation in these loci is also correlated with phenotypic variation between breeds. An alternative approach, when a particular trait is shared by several breeds, is to perform across-breed GWAS. In general, levels of LD decay much faster between breeds, and this reduces the power to detect association [11]. However, selection acts to fix long haplotypes bearing the causative variant, thus increasing levels of LD between breeds in regions under selection. Jones *et al.* [12] used a sparse marker set and across-breed GWAS to identify correlations with a number of morphological traits, such as size, height, and shape of ears, snout and limbs, which was further refined by Boyko *et al.* [6] using 80 dog breeds and ∼61,000 SNPs. Across-breed GWAS have also been used to identify an FGF4 retrogene associated with chondrodysplasic breeds [13] and the THBS2 locus associated with brachycephalic breeds [14].

Genomic regions with a high degree of genetic differentiation between breeds are also indicative of selection. A large proportion of SNPs with high *F_ST_* between dog breeds are found in loci associated with phenotypic traits such as size, ear morphology and coat color [6]. Akey *et al.* [15] scanned patterns of variation in 10 dog breeds and ∼21,000 SNPs using a 1 Mb sliding windows to identify larger regions with elevated *F_ST_* in particular breeds. This scan identified many regions likely to be under selection in one or more of the breeds in their dataset. Notably, a highly differentiated interval in Shar-Pei on chromosome 13 contains the HAS2 gene and is likely associated with the wrinkled skin phenotype of this breed [15], [16].

Although a large number of loci under selection have now been identified, the genetic basis of much of the phenotypic variation in dog breeds and particularly behavioral traits remains unexplained. One drawback of previous studies is the use of SNP arrays with relatively low coverage of the genome. With the development of a new high-density array it is now possible to examine the dog genome at much higher resolution, allowing a comprehensive characterization of regions under selection. Genetic variants under selection in dogs can be loosely divided into two categories: 1) those that control variation in common traits such as size and ear carriage, which segregate across many breeds [6], [8] and 2) those that encode rare traits that present in one or a few breeds, such as brachycephaly, chondrodysplasia and skin wrinkling [13], [14], [16].

Here we implement a variety of approaches to identify both these types of loci. In cases where a common trait has been identified, it is possible to search for genotype-phenotype correlations. We attempt to identify both behavioral and morphological traits that vary between breeds using across-breed GWAS. We also use *F_ST_* statistics to identify additional SNPs that have high variability in frequency between breeds. These methods identify known loci and indicate new regions that may be involved in common trait variation.

The action of selection can potentially be identified by examining patterns of variation in individual breeds in order to detect the characteristic signature of selective sweeps. This signature is characterized by the presence of long haplotypes, a skew in allele frequency, reduced heterozygosity, and elevated population differentiation. A large number of statistical methods have been developed to detect sweeps based on these different patterns [17]-[22]. The formation of dog breeds occurred during an extremely brief evolutionary time, and likely involved rapid fixation of haplotypes under strong artificial selection. Under this scenario, simulations suggest that statistics based on *F_ST_* and differences in heterozygosity are likely to be most powerful. [23]. Furthermore, dog breeds are known to be characterized by extensive LD and limited haplotype diversity, including long blocks of homozygosity, which reflect the action of population bottlenecks and selective breeding. This suggests that tests based on allele frequency spectrum and haplotype length will be of limited applicability, as many genomic regions are essentially devoid of genetic variation. We therefore base our approach to identify selective sweeps on pairwise comparisons of both *F_ST_* and heterozygosity between breeds.

The presence of long blocks of homozygosity in the dog genome [1], [11] is likely to reflect the action of both selection and genetic drift. We therefore conduct extensive coalescent simulations in order to distinguish between these processes. These simulations incorporate a realistic model of dog population history under neutrality to provide null distributions to compare with the real data. We also conduct a comprehensive characterization of SNP variation in a 3 Mb region encompassing several loci with extreme population differentiation that are associated with at least two morphological traits.

## Results

### High-density canine array design and evaluation

Our first goal was to develop a high-density, high-accuracy mapping array with uniform SNP coverage across the whole genome. Since the SNP map from the canine genome project, although containing >2.8 million SNPs at fairly even coverage, still contained gaps, we first performed targeted resequencing within 1,555 regions that lie within intervals >40 kb containing no known SNPs in unique sequence. We performed Roche NimbleGen array capture to enrich these regions followed by sequencing using the Illumina Genome Analyzer on 4 pools containing multiple samples of a single dog breed (Irish Wolfhounds, West Highland White Terrier, Belgian Shepherds and Shar-Pei) and one pool of wolf samples. In total, we discovered 4,353 additional high-quality SNPs using this method. We selected SNPs from this improved map to form the “CanineHD” array panel. We generated an initial panel of 174,943 SNPs that were included on the array of which 173,622 (99.2%) give reliable data. These loci are distributed with a mean spacing of 13 kb and only 21 gaps larger than 200 kb. Loci with unreliable SNP calls, potentially due to copy number polymorphism, were not included in the analysis. In total, 172,115 are validated for SNP genotyping and 1,547 are used only for probe intensity analyses. This is a significant improvement compared with the largest previously existing array, which has 49,663 well performing SNPs, with a mean spacing of 47 kb and 1,688 gaps larger than 200 kb. Figure S1 shows the distribution of SNPs in 100 kb windows across the genome. The improvement in coverage is particularly striking on the X chromosome, where >75% of 100 kb windows contain no SNPs on the previous array, but <5% of windows do not contain SNPs on the CanineHD array.

Of all the SNPs on the array, 0.9% are novel SNPs discovered by the targeted resequencing experiment. The remaining SNPs have been previously described: 65.1% of them were present in a comparison of the boxer reference genome with a previously sequenced poodle, 21.7% were present in alignments of low coverage sequencing reads from various dog breeds to the boxer reference genome, 25.4% were present within the boxer reference and 1.2% were present in alignments of wolf and/or coyote sequencings with the reference boxer genome. There is therefore a bias in the way that SNPs were ascertained: all of them were identified in a comparison involving the boxer reference assembly. However this has not had a great impact on the number of SNPs polymorphic in different breeds (see below). The array was initially evaluated using 450 samples from 26 breeds termed the “Gentrain” dataset. Within this dataset, average call rates were 99.8% and reproducibility and Mendelian consistency were both >99.9%. A subset of 24 samples generated by whole genome amplification (WGA) of 12 blood and 12 cheek swab samples produced slightly lower call rates (blood-WGA 99.3%; buccal-WGA 98.9%). Probe intensities from the array can also be used to analyze copy number polymorphisms, although this is not evaluated here.

### Dataset construction

To perform a broader analysis of canine breed relationships and selective sweeps, we constructed a larger dataset consisting of unrelated samples from the Gentrain dataset, and unrelated control dogs genotyped for disease gene mapping studies from multiple breeds as part of the LUPA consortium. This dataset, which we refer to here as the “full LUPA genotype dataset” consists of 509 dogs from 46 diverse breeds and 15 wolves, genotyped on the CanineHD array. These include 156 dogs from 13 breeds derived from LUPA control dogs and 353 dogs from 33 breeds from the Gentrain dataset (See Table S1 for full details). A subset of this dataset, referred to here as the “reduced LUPA genotype dataset” is made up of all the samples in the 30 breeds (plus wolf) with more than 10 samples in the full dataset (471 samples in total).

Table 1 shows patterns of polymorphism in the reduced LUPA genotype dataset. In total, 157,393 SNPs on the array were polymorphic (90% of SNPs on the array). A mean of 119,615 SNPs (69%) were polymorphic within a single dog breed. Hence although there is a bias in the way that SNPs were ascertained, there is a substantial amount of variation within all breeds surveyed. On average 39 SNPs were polymorphic only in one breed, although this figure shows large variation between breeds. A subset of 1,471 SNPs were variable in wolves but not within any dog breed. However, most of these SNPs were originally discovered by comparisons of sequences from different dog breeds, which suggests that they are also variable between (but not within) dog breeds.

**Table 1:** Levels of genetic variation in breeds with 10 or more samples.

Breed	Abbreviation	No. Samples	Seg. sites	Private seg. sites
Belgian Tervuren	BeT	12	115,154	0
Beagle	Bgl	10	115,254	16
Bernese Mountain Dog	BMD	12	106,152	15
Border Collie	BoC	16	127,491	7
Border Terrier	BoT	25	108,344	15
Brittany Spaniel	BrS	12	130,115	11
Cocker Spaniel	CoS	14	126,118	19
Dachshund	Dac	12	131,372	5
Doberman Pinscher	Dob	25	112,627	19
English Bulldog	EBD	13	111,720	19
Elkhound	Elk	12	127,066	82
English Setter	ESt	12	121,196	24
Eurasian	Eur	12	120,360	6
Finnish Spitz	FSp	12	109,510	20
Gordon Setter	GoS	25	134,615	12
Golden Retriever	Gry	11	112,144	10
Greyhound	GRe	14	128,907	45
German Shepherd	GSh	12	108,614	11
Greenland Sledge Dog	GSl	12	102,899	19
Irish Wolfhound	IrW	11	92,718	61
Jack Russell Terrier	JRT	12	137,837	12
Labrador Retriever	LRe	14	129,951	23
Newfoundland	NFd	25	127,503	13
Nova Scotia Duck Tolling Retriever	NSD	23	118,387	36
Rottweiler	Rtw	12	107,022	15
Schipperke	Sci	25	126,530	21
Shar-Pei	ShP	11	124,828	93
Standard Poodle	StP	12	132,289	123
Yorkshire Terrier	TYo	12	129,768	388
Weimaraner	Wei	26	111,958	21
Wolf	Wlf	15	118,256	1,471
Total	-	471	157,393	-

### Evolutionary relationships between dog breeds

**Figure 1:**
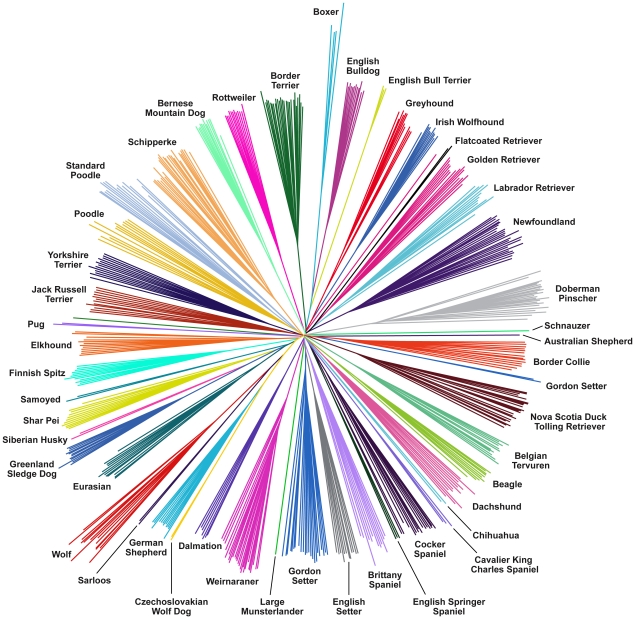
Neighbor-joining tree constructed from raw genetic distances representing relationships between samples. More than 170,000 SNPs were genotyped in 46 diverse dog breeds plus wolves using the CanineHD array. The boxer branches are longer, which likely represents the influence of ascertainment bias, as the SNPs were discovered from sequence alignments involving the boxer reference sequence.

We used the CanineHD array to investigate breed relationships by constructing a neighbor-joining tree [24] of raw genetic distances in the full LUPA genotype dataset (Figure 1). Three main features are obvious: 1) Dogs from the same breed almost invariably cluster together. This reflects the notion that modern breeds are essentially closed gene pools that originated via population bottlenecks. 2) Little structure is obvious in the internal branches that distinguish breeds. This is consistent with the suggestion that all modern dog breeds arose from a common population within a short period of time and that only a very small proportion of genetic variation divides dog breeds into subgroups. 3) The internal branches leading to boxer and wolf are longer than those leading to other breeds. The long boxer branch can be explained by the fact that a large proportion of the SNPs were assayed by comparing boxer with other breeds, which implies that the dataset is enriched for SNPs that differ between boxer and other breeds. The longer wolf branch probably reflects more distant relatedness.

Some breeds show a tendency to group together in the tree, such as breeds of retrievers, spaniels, setters, and terriers. However, the length of the internal branches leading to these clusters is only a small fraction of the average total length of branches in these clusters, which indicates that genetic variation in dogs is much more severely affected by breed creating bottlenecks than it is by historical origins of various breeds, although detailed analysis of these data has power to reveal their historical origins [25]. The most obvious clustering of breeds is exhibited by two wolf hybrids: Sarloos and Czechoslovakian wolf dog, which exhibit a closer relationship to the wolf than other breeds as predicted by their known origin [26]. The German shepherd also clusters with this group, although this is likely to be a result of its close relationship with the Czechoslovakian wolf dog, rather than with wolf. The tree is consistent with previous studies and supports the accuracy and reliability of the array. Although the long boxer branch likely reflects SNP ascertainment bias on the array, the tree reflects extensive polymorphism both within and between breeds. This suggests the SNP ascertainment scheme is not problematic and that the array is well suited for both within and across breed gene mapping.

We performed coalescent simulations modeling the ascertainment bias, sample size, and inferred recombination rate in the true dataset (see Materials and Methods) in order to predict the expected patterns of genetic diversity that we expect to observe within and between breeds in the absence of selection. The bottleneck population sizes at breed creation used in the simulations are presented in Table S2. The decay of LD in the simulated data closely matches the real decay in LD (Figure S2).

### Across-breed GWAS: morphological traits

To identify genetic variation associated with common traits that vary among breeds, we performed across-breed GWAS using the full LUPA genotype dataset. A list of traits and their variation between breeds is in Table S3. Each sample was given a value corresponding to the standardized breed phenotype for the trait under study. We performed quantitative association studies for size and personality traits whereas other traits were binary coded. For each GWAS, we assayed genome-wide significance by permuting the phenotype of each breed, assigning each dog of the same breed with identical phenotype values. The true significance of genotype-phenotype correlation at each SNP was compared with the maximum permuted value of all SNPs across the array in order to estimate genome-wide significance (see Materials and Methods). This permutation procedure corrects for the extreme population substructure present in dog breeds.

Using this method we were able to replicate several known associations. We first performed a GWAS comparing 4 breeds with furnishings (a coat type with moustache and eyebrows [9]) compared to 42 without them. Genome-wide significant associations were observed at 3 SNPs distributed located between 10.42 - 11.68 Mb on chromosome 13. The most strongly associated SNP is at 11,678,731 (P_genome_<0.001), 44 kb from the causative SNP previously identified in RSPO2 [9]. We next scanned the genome for associations with size, using weight in kilograms as a proxy (data taken from [8]; see Table S3). The most strongly associated SNP was located on chromosome 15 at 44,242,609 (P_genome_ = 0.004), which is within the IGF1 gene, previously implicated in size variation [8]. Genome-wide significant associations (P_genome_<0.05) were observed at 7 SNPs within an interval between 44.23 - 44.44 Mb. In addition, we observed an association within a previously defined region on chr10 (11,169,956 bp; P_genome_ = 0.036). The SNP at chr10:11,169,956 is about 500kb upstream of HMGA2, which has been established to be associated with body size variation in other species [27]–[29].

The frequency of the SNP (chr15:44,242,609) most strongly associated to size shows a steady decline according to the size of the breed. However, the differences in allele frequency at the SNP chr10:11,169,956 are more striking, as one allele appears at very low frequencies in all breeds apart from a number of very small breeds (Yorkshire Terrier, Border Terrier, Jack Russell Terrier, Schipperke), where it is at or close to fixation (Figure S3). Hence, there appears to be relatively continuous variation in frequency in a variant affecting IGF1 between breeds, whereas a variant upstream of HMGA2 appears to have been fixed in a subset of small breeds but shows little variation in allele frequencies in other breeds.

**Figure 2:**
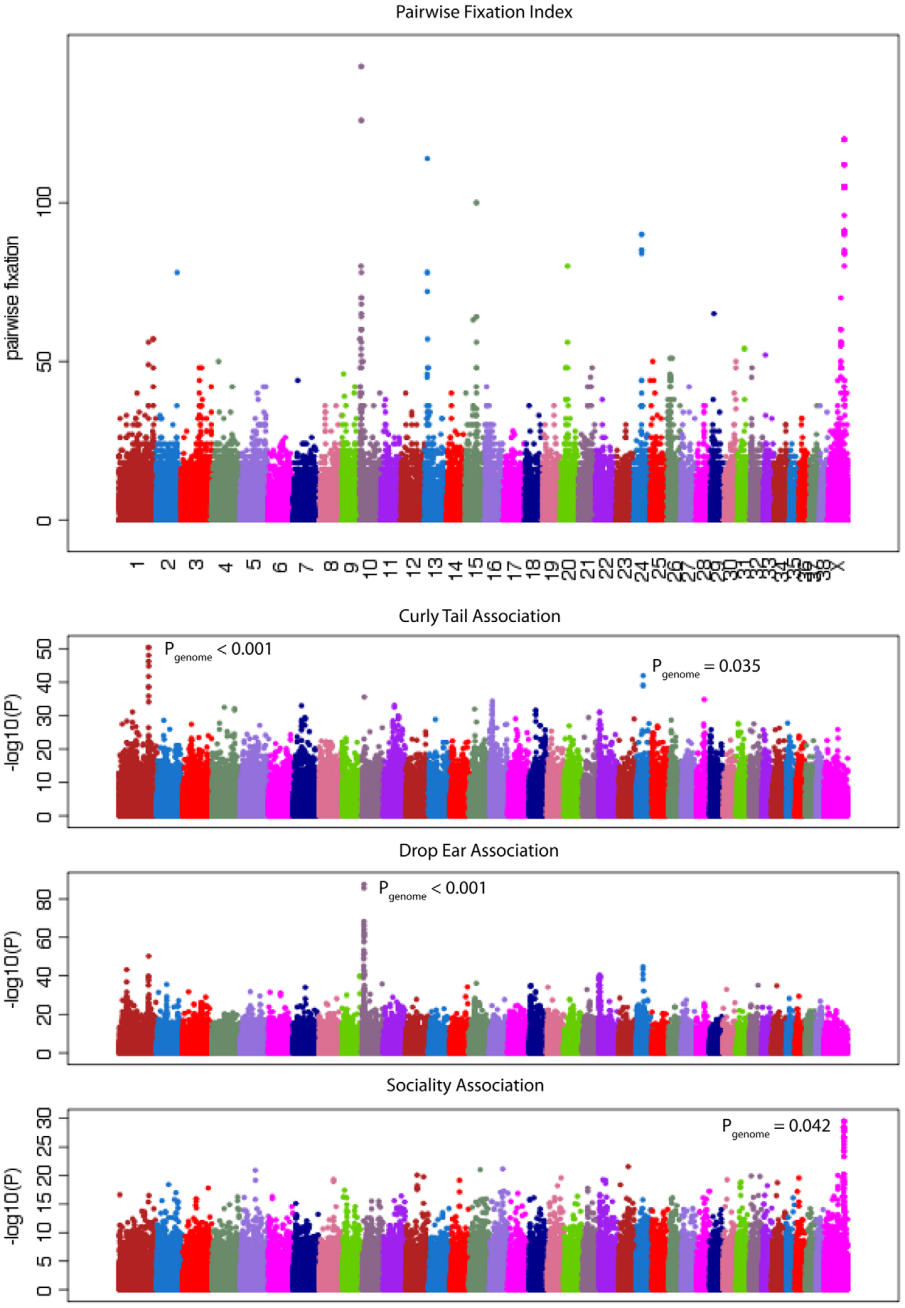
Identification of variants with large differences in allele frequencies between breeds that are associated with phenotypic variation. The top panel shows the variation in pairwise fixation index (see text for definition) at SNPs across the genome on the CanineHD array. The bottom panel shows GWAS for three traits (curly tail, drop ear, and sociality) with signals that correspond to SNPs with high population differentiation. P-values from breed permutations are also shown.

Dog breeds show extreme variation in ear morphology ranging from pricked ears to low hanging dropped ears. We performed a GWAS using 12 breeds with pricked ears and 15 breeds with dropped ears. Within an interval between 10.27 - 11.79 Mb, 23 SNPs had genome-wide significant associations (P_genome_<0.05; Figure 2). The most strongly associated SNP was chr10: 11,072,007 (P_genome_<0.001), which lies between the HGMA2 and MSRB3 genes. This region has been associated with ear type and body size in previous studies [6], [12]. Using the CanineHD array, we are able to type SNPs at a much higher density in the associated region. There is also large variation between dog breeds in degree of tail curl. We classified breeds in our dataset into 11 with curly tails and 7 with straight tails and performed a GWAS. Six SNPs on chromosome 1 were most significantly associated within an interval 96.26 - 96.96 Mb (P_genome_<0.05; Figure 2), which are downstream of RCL1 and upstream of JAK2 (Figure S4). This region has not been previously associated with tail curl.

### Across-breed GWAS: behavioral traits

We performed GWAS to search for variants that affect breed differences in behavior. We first performed a GWAS by comparing 18 bold and 19 non-bold breeds using phenotypic definitions from ref. [12]. Highly significant associations were found at two SNPs on chromosome 10, 11,440,860 (P_genome_<0.001) and 10,804,969 (P_genome_ = 0.006), in the same region associated with both drop ear and size. Variation within this region is therefore associated with at least two morphological and one behavioral trait, which may be correlated. The region contains several genes including WIF1, HMGA2, GNS, and MSRB3 (see Figure S5). However, the most significant associations for each trait appear to occur in different places. The SNPs most associated with drop ear and size occur 98 kb apart between the MSRB3 and HMGA2 genes, with the drop ear association closer to MSRB3, whereas the top boldness association occurs within an intron of HMGA2, 271 kb 3′ of the size association. There is however a strong correlation between the bold and non-bold breed classifications and the drop ear and size classifications. All prick eared and small dogs were classified as bold in the dataset, whereas all drop eared dogs were classified as non-bold, with the exception of Bernese Mountain Dog (see Table S3).

Breed averages for five personality traits measured objectively under controlled conditions were obtained from the Swedish Kennel Club. The traits are defined as sociability, curiosity, playfulness, chase-proneness and aggressiveness [30] and have been shown to be consistent among multiple tests of the same dog [31]. We performed quantitative GWAS using the breed-average trait values presented in Table S3. We observed significant associations at a number of SNPs for the trait sociability, which measures a dog's attitude toward unknown people (Figure 2). No SNPs reached genome-wide significance, but a large number of SNPs on the X chromosome also showed strong association. In order to accurately measure genome-wide significance in the sex chromosomes compared to autosomes we removed male dogs from the analysis. This analysis identified 10 SNPs with genome-wide significant associations (P_genome_<0.05) in the interval 106.03–106.61 Mb on the X chromosome (see Figure S6). This region was also identified in a previous study [6] to be highly differentiated between breeds and correlated with body size and skull shape.

### Single-SNP F_ST_ statistics identify SNPs under selection in multiple breeds

Across breed GWAS is a powerful approach for identifying genotype-phenotype relationship for traits shared among breeds. The variants identified by this approach, by definition, have large variation in allele frequencies between breeds. However, there may be many more such SNPs that have been subjected to similar selective pressures for common traits between breeds where the trait is not identified. In order to find such loci, we identified SNPs that exhibit high levels of differentiation between dog breeds using the *F_ST_* statistics calculated for the >173,000 SNPs in the reduced LUPA genotype dataset.

**Figure 3:**
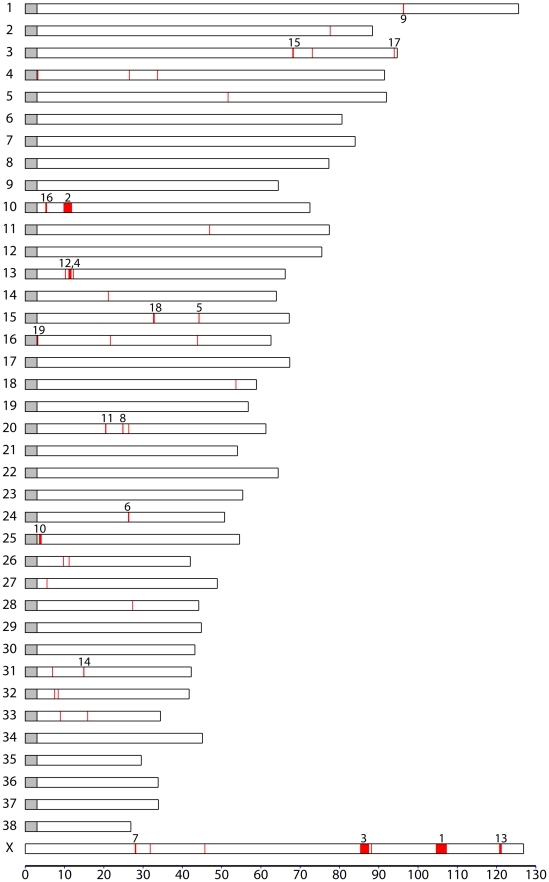
Map of regions with extreme differentiation between dog breeds as identified by single-SNP *F_ST_*. All regions with at least one SNP with *F_ST_* >0.55 and minor allele frequency >15% are shown. Numbers correspond to the regions in Table 2, which contain at least two nearby SNPs that pass these thresholds.

A total of 240 SNPs have a *F_ST_*>0.55 and overall minor allele frequency >0.15 in the reduced dataset containing breeds with at least 10 samples. These cut offs are identical to those used by Boyko et al. [6] and are chosen for comparison. In the simulated data, no SNPs pass this cut off (p<0.0001; χ^2^ test). We then generated a list of highly differentiated regions, by merging all SNPs in this list within 500kb of each other into single regions, resulting in 44 regions containing between 1 and 94 SNPs with elevated *F_ST_*. Regions with two or more SNPs are presented in Table 2 and the complete list is presented in Table S4. Figure 2 presents a value for each SNP (used for illustration purposes only) that we term “pairwise fixation index” to highlight differences in allele frequencies between breeds. This is defined as *pq*, where *p* is the number of breeds where allele A is fixed or close to fixation (frequency >0.95) and *q* is the number of breeds where allele B is fixed or close to fixation (frequency >0.95). In total 53,944 out of 154,034 variable SNPs have a *pq* value > 0, indicating that they are fixed for different alleles in at least 2 breeds. The regions of high *F_ST_* correspond strongly to loci where trait associations have been reported. In particular, 8 of the 9 regions comprised of more than 3 high-*F_ST_* SNPs overlap known trait-associated regions, and it is likely that most or all of the remaining regions with high *F_ST_* show a correlation with an as yet undefined trait. Three of these regions were not previously reported in a study based on a less dense array [6] including a region on chromosome 7 (27.99 - 28.15 Mb) containing five highly differentiated SNPs that encompasses the DMD gene. The locations of all regions are marked in Figure 3, which presents a comprehensive map of regions that are likely to contain major loci influencing phenotypic variation between dog breeds.

**Table 2:** Description of regions with at least two nearby SNPs with high ***F_ST_*** (>0.55) and high minor allele frequency (>15%).

no.	chr	start (bp)	end (bp)	no. SNPs	length (kb)	max *F_ST_*	association	candidate genes
1	X	104,640,567	107,235,825	96	2,595	0.75	sociality*, size, skull shape	
2	10	9,836,009	11,792,711	33	1,957	0.81	drop ear, size, boldness*	WIF1, HMGA2, MSRB3
3	X	85,365,233	87,444,776	29	2,080	0.58	limb/tail length	
4	13	11,095,120	11,678,731	10	584	0.73	furnishings	RSPO2
5	15	44,216,576	44,267,011	6	50	0.68	size	IGF1
6	24	26,270,399	26,370,499	5	100	0.70	coat color	ASIP
7	X	27,990,332	28,152,042	5	162	0.63	*	DMD
8	20	24,841,077	24,889,547	4	48	0.63	coat color	MITF
9	1	96,286,007	96,335,577	4	50	0.58	snout ratio, curly tail*	RCL1
10	25	3,603,872	4,065,978	3	462	0.63	*	FOXO1, BRD2
11	20	20,449,477	20,539,359	3	90	0.62	*	KLF15, ZXDC, UROC1, TXNRD3
12	13	10,210,459	10,225,305	3	15	0.59	*	OXR1
13	X	120,769,286	121,212,627	2	443	0.70	*	MAGEA, THEM185A
14	31	14,888,449	14,944,938	2	56	0.61	*	NRIP1
15	3	68,103,223	68,260,652	2	157	0.60	*	CPEB2
16	10	5,221,427	5,440,236	2	219	0.60	size*	CDK4
17	3	93,933,450	93,944,095	2	11	0.60	size	LCORL
18	15	32,638,117	32,853,840	2	216	0.57		KITLG
19	16	3,198,732	3,212,612	2	14	0.56	*	PKD1L1

Associations (or regions with no suggested association) marked with an asterisk are novel to this study. Others are summarized in [6].

Three regions longer than 1Mb are identified by this measure, likely signifying regions under strong selection in many breeds. These consist of a 2.6 Mb region on chromosome X that associates with body size, skull shape and sociability, a 2.0 Mb region on chromosome 10 that associates with drop ear, size and boldness and a 2.1 Mb region on chromosome X associated with limb and tail length (see also [6]). Other loci identified include three loci involved in coat type (RSPO2, FGF5, KRT71) [9]. In particular the RSPO2 gene associated with furnishings is found within an extended 0.6 Mb region. The MITF and ASIP (Agouti) genes known to be involved in coat color in dogs [11] are also identified. The region on chromosome 1 identified here as associated with curly tail and previously associated with snout ratio [6] is associated with 4 SNPs with high *F_ST_* across 50 kb. Other genes of note identified are LCORL, known to associate with human height [27], [29], KITLG, associated with coat color in other species [32] and several genes with key developmental roles, such as sonic hedgehog (SHH) involved in patterning in the early embryo, msh homeobox 1 (MSX1), involved in embyrogenesis and bone morphogenic protein 1 (BMP1) involved in bone development.

### Genome-wide scans for signatures of selective sweeps in single breeds

Rare selective sweeps corresponding to regions of the genome under selection in only one or a small number of breeds in our dataset cannot be detected by across-breed GWAS due to lack of power. They also have a weak effect on *F_ST_* values at single SNPs across all breeds compared to regions under selection in many breeds. In order to identify such rare sweeps, we scanned patterns of variation in the reduced LUPA genotype dataset to identify extended regions where haplotypes had become fixed in one or more breeds, leading to a local reduction in genetic variation and increase in population differentiation. We analyzed 150 kb sliding windows, overlapping by 25 kb in each breed compared with other breeds using two statistics. The first statistic, *S_i_*, is calculated by summing regional deviations in levels of relative heterozygosity across the genome between two breeds compared to the genomic average and summing across all pairwise comparisons. Relative heterozygosity is defined as the number of SNPs segregating in a genomic window in one breed divided by the number of SNPs segregating in that window in two breeds under comparison. Hence, regions with low *S_i_* in a breed contain few segregating SNPs compared to other breeds. The second statistic, *d_i_*, was implemented by Akey et al. [15], and is based on pairwise *F_ST_* values normalized for a given breed relative to the genome-wide average, summed across all pairwise combinations involving the given breed. Regions of high *d_i_* in a particular breed exhibit a large difference in allele frequencies compared with other breeds.

We first identified windows with *S_i_* or *d_i_* values in an extreme 1% tail of their respective distributions (the bottom 1% for *S_i_* and top 1% for *d_i_*). Overlapping windows were then collapsed into larger regions (see Materials and Methods). These regions represent a map of blocks of reduced heterozygosity or elevated population differentiation in each breed. We repeated our analysis of *d_i_* and *S_i_* on the simulated data (see above). For both statistics, the average length of regions identified was similar in real versus simulated datasets. However, there was a strong excess of regions >250 kb in the real compared with simulated datasets, which likely reflects regions influenced by selection. In order to distinguish regions generated by genetic drift compared with those generated by selective sweeps we first estimated a marginal p-value for each block, equal to the proportion of simulated blocks with longer lengths in the same breed. We then adjusted these p-values using a 5% False Discovery Rate (FDR; see Materials and Methods and ref. [33]). In total 524 high confidence putative sweeps (an average of 17 per breed) were identified using the *S_i_* statistic, with a mean size of 475 kb. However, none of the regions identified by the *d_i_* statistic remained significant after FDR correction. Figure S7 shows the distribution of significant *S_i_* regions in the dog genome.

Full lists of regions identified by the *S_i_* and *d_i_* analyses including the marginal and FDR corrected p-values are presented in Table S5 and summary statistics of these regions are presented in Table S6. These regions are also available as a UCSC annotation dataset (see Materials and Methods for URLs). The UCSC browser offers a graphical display of *S_i_* and *d_i_* regions as well as *d_i_* values for all SNPs analyzed [34]. Table S7 shows the overlap between these regions and those identified in previous studies (refs. [6] and [15]).

### Long regions of reduced heterozygosity are identified by the *S_i_* statistic

The *S_i_* test identifies blocks of the genome where one breed has little or no variation consistent with fixation of a long haplotype by a selective sweep. On average, only 19.9% of SNPs have segregating variants in these regions in the breed where they are identified compared with the genome average of 74.5%. Among the 524 putative sweeps are several loci already implicated in breed-defining characters. Notably, a 590 kb region of low *S_i_* overlapping the FGF4 retrogene on chromosome 18 associated with chondrodysplasia in Dachshunds. A 1.4 Mb region of low *S_i_* overlapping the HAS2 gene implicated in skin wrinkling [16] is observed in Shar-Pei. Regions in the vicinity of the RSPO2 locus implicated with furnishings are observed in 2 breeds, which both have furnishings (Yorkshire Terrier and Standard Poodle). However, many variants implicated in phenotypic variation between breeds are not strongly associated with regions of reduced *S_i_*. No putative sweeps overlapping the IGF1 locus are identified in small breeds using this statistic. This is likely to be because there appears to be continuous variation in allele frequency at this locus between breeds rather than complete fixation of certain haplotypes in several breeds (see Figure S3).

Table 3 shows the top 20 longest regions of significantly reduced *S_i_*. It should be noted that two pairs of putative sweep regions occur at contiguous locations in the same breed (no. 2 and 12 in Beagle and no. 5 and 6 in Irish Wolfhound), which could potentially represent single selective sweeps. The longest region we identified is 3.1 Mb long (chr22: 5.3–8.4 Mb) in beagles. This region overlaps 3 other putative sweeps within the top 20 in other breeds (Gordon Setter, Rottweiler, and Newfoundland) whereas no other regions in the top 20 are overlapping. As this and other regions with strongest evidence for sweeps are long and contain many genes, it is not possible to identify the locus under selection in a single sweep. However, it is interesting to note that they contain genes associated with disease in humans and dogs including epilepsy (KCNQ5), cancer (NPM1, FGR), and autoimmune disease (IL6). A long sweep on chromosome 30 in Golden retrievers spans the RYR1 gene, involved in the skeletal muscle calcium release channel and implicated in canine malignant hyperthermia by linkage analysis [35]. We also identified a number of genes involved in spermatogenesis and fertilization (SPAG1, FNDC3A, CLGN) which is a category often enriched in genes under positive selection in other species [36].

**Table 3:** Description of longest regions with significant drop in *S_i_*.

no.	breed	chr	start	end	length	no. genes	overlaps
1	Bgl	22	5,286,218	8,423,791	3,137,574	27	*S_i_* (1,11,13,17) *d_i_* (1,8,9)
2	EBD	26	8,813,638	11,587,844	2,774,207	70	*d_i_* (7,14)
3	ESt	25	27,590,228	30,101,489	2,511,262	29	
4	ShP	13	3,036,181	5,264,386	2,228,206	24	*d_i_* (3,12)
5	IrW	2	21,323,001	23,188,692	1,865,692	18	
6	IrW	2	19,483,009	21,068,544	1,585,536	20	
7	Bgl	14	39,147,833	40,601,429	1,453,597	22	
8	GRe	30	3,901,787	5,329,451	1,427,665	12	
9	ShP	13	23,047,599	24,433,840	1,386,242	6	
10	Gry	25	7,271,327	8,636,861	1,365,535	10	
11	GoS	22	4,904,331	6,215,828	1,311,498	14	*S_i_* (1,11,13,17) *d_i_* (1,8,9)
12	EBD	26	12,121,983	13,432,228	1,310,246	24	
13	Rtw	22	4,654,448	5,937,680	1,283,233	18	*S_i_* (1,11,13,17) *d_i_* (1,8,9)
14	Gry	19	4,473,961	5,756,046	1,282,086	10	
15	BMD	2	75,308,805	76,571,807	1,263,003	35	
16	Bgl	12	37,413,405	38,655,096	1,241,692	13	
17	NFd	22	4,752,012	5,980,115	1,228,104	17	*S_i_* (1,11,13,17) *d_i_* (1,8,9)
18	Rtw	13	50,613,526	51,755,732	1,142,207	16	
19	Dob	20	39,397,583	40,517,365	1,119,783	34	
20	GRe	8	5,436,616	6,533,588	1,096,973	42	

In cases where multiple breeds are affected by selection acting on the same variant, it may be possible to narrow an interval containing the causative mutation by identifying a core region of identity by state (IBS) between all breeds where haplotypes are shared, most likely reflecting common ancestry. We searched our dataset for regions with significant drops in *S_i_* that overlapped between different breeds. We then identified the maximal region where the same haplotype was fixed in all breeds identified. For many significant long regions we were able to identify shorter regions of IBS. The regions shared by 3 or more breeds are shown in Table 4 and a full list is in Table S5. As a validation of this method, we identified a 187 kb region where an identical haplotype is fixed among the 3 breeds with furnishings where we identified a sweep spanning the previously defined causative indel (region 14). Hence this method is able to identify interval containing the causative mutation in shared region of identity by descent.

**Table 4:** Description of regions with identical fixed haplotypes across multiple breeds.

no.	chr.	start	end	length	no. breeds	no. genes	candidate genes
1	22	5,466,185	5,950,731	484,547	8	2	FNDC3A,CYSLTR2
2	37	3,453,815	3,830,321	376,507	7	6	MSTN
3	X	101,638,881	101,992,724	353,844	5	1	UBE2I
4	6	26,881,144	27,285,067	403,924	4	7	CRYM,ZP2
5	1	7,427,961	7,811,190	383,230	4	2	ZNF407
6	23	5,694,045	5,971,463	277,419	4	4	HSP90AA1
7	21	4,828,296	5,034,632	206,337	4	1	CNTN5
8	17	3,190,961	3,672,468	481,508	3	4	TMEM18
9	19	5,046,934	5,463,342	416,409	3	7	UCP1
10	2	80,709,265	81,050,769	341,505	3	2	EIF4G3
11	37	14,809,394	15,087,734	278,341	3	6	NBEAL1
12	X	112,830,694	113,040,282	209,589	3	3	ATP11C
13	11	49,319,964	49,523,469	203,506	3	1	LINGO2
14	13	11,509,194	11,695,899	186,706	3	1	RSPO2
15	12	36,463,722	36,557,249	93,528	3	0	B3GAT2
16	8	24,377,123	24,415,610	38,488	3	0	CCT6P1

The inferred selective sweep shared by the most breeds in this analysis was a 485 kb haplotype on chromosome 22 (5.4–5.9 Mb) shared by 8 breeds (Beagle, Border Terrier, English Bulldog, Gordon Setter, Irish Wolfhound, Newfoundland, Rottweiler, Weimaraner). This region contains 2 genes: FNDC3A, fibronectin type III domain containing 3A [37], which is involved in spermatogenesis and also expressed in odontoblasts indicating a role in odontogenesis, and CYSLTR2 cysteinyl leukotriene receptor 2, a member of the superfamily of G protein-coupled receptors. A 402 kb haplotype on chromosome 37 (3.5–3.8 Mb) is shared among 7 breeds (Bernese Mountain Dog, Beagle, Border Terrier, Doberman, Elkhound, Finnish Spitz, Golden Retriever). This haplotype contains 7 genes including the MSTN (myostatin) gene. This gene is associated with double muscling in cattle [38] and in a similar phenotype observed in whippets [39]. It is therefore plausible that this region has been a target of selection in multiple dog breeds in order to modify muscle mass. A 354 kb haplotype on chromosome X is fixed in 5 breeds (101.6–102.0 Mb) and contains only one gene: UBE2I, an ubiquitin-conjugating enzyme. This enzyme has been shown to interact with MITF, involved in coat color, and is suggested to be a key regulator of melanocyte differentiation [40] although it also has a number of other features.

### Regions of elevated population differentiation are identified by the *d_i_* statistic

There are many extremely differentiated regions although none of them passed the 5% FDR correction for length (see Table S5 for full list). Variation in *S_i_* and *d_i_* statistics in the 10 longest regions identified by the *S_i_* test is presented in Figure S8. This comparison of the *d_i_* and *S_i_* tests reveals that the increases in *d_i_* often occur within a more restricted region of a large block of fixed haplotype from the *S_i_* tests, indicating that they represent regions where an otherwise rare ancestral sub-haplotype has been fixed in a certain breed. It therefore appears that many regions detected by *d_i_* and *S_i_* tests are complementary. Among the top 20 longest putative sweeps identified by the *d_i_* statistic (Table 5) are 3 overlapping sweeps that also overlap the common sweep containing FNDC3A and CYSLTR2 identified by the *S_i_* test. We also identify putative sweeps in 4 breeds overlapping the region associated with drop ear, size and boldness among the top 20 *d_i_* sweeps. Two putative sweeps in this list overlap a region on chromosome 13 (3.3–5.2 Mb), which is also identified by the *S_i_* test. One gene of note in this region is VPS13B, which may have an important role in development and is associated with Cohen syndrome, which has an effect on development of many parts of the body [41]. The second longest putative sweep identified by *S_i_* on chromosome 26 is also identified in two of the top 20 longest *d_i_* regions.

**Table 5:** Description of the longest regions with elevated *d_i_*.

no.	breed	chr	start	end	length	no. of genes	overlaps
1	ShP	22	4,828,721	6,233,045	1,404,325	15	*d_i_* (1,8,9) *S_i_* (1,11,13,17)
2	JRT	10	10,265,925	11,644,756	1,378,832	10	*d_i_* (2,4,10,18)
3	Elk	13	3,778,724	5,152,585	1,373,862	12	*d_i_* (3,12) *S_i_* (4)
4	TYo	10	10,265,925	11,559,700	1,293,776	8	*d_i_* (2,4,10,18)
5	EBD	9	4,125,282	5,418,477	1,293,196	22	
6	BoC	22	15,982,263	17,193,960	1,211,698	2	
7	EBD	26	10,491,787	11,629,631	1,137,845	29	*d_i_* (7,14) *S_i_* (2)
8	FSp	22	4,300,860	5,397,353	1,096,494	19	*d_i_* (1,8,9) *S_i_* (1,11,13,17)
9	NFd	22	4,300,860	5,361,506	1,060,647	18	*d_i_* (1,8,9) *S_i_* (1,11,13,17)
10	Elk	10	10,707,193	11,644,756	937,564	7	*d_i_* (2,4,10,18)
11	LRe	13	40,738,270	41,665,437	927,168	36	
12	Bgl	13	3,266,021	4,176,521	910,501	13	*d_i_* (3,12) *S_i_* (4)
13	TYo	3	40,471,308	41,367,554	896,247	11	
14	TYo	26	10,744,458	11,629,631	885,174	23	*d_i_* (7,14) *S_i_* (2)
15	ESt	19	46,765,322	47,637,012	871,691	5	
16	ShP	3	3,069,417	3,922,440	853,024	4	
17	TYo	24	25,532,482	26,370,499	838,018	20	
18	BrS	10	10,832,919	11,644,756	811,838	5	*d_i_* (2,4,10,18)
19	BoT	19	10,239,039	11,031,247	792,209	3	
20	IrW	37	3,170,534	3,960,864	790,331	12	

### XP-EHH

We performed an additional validation of our results using a third statistic, XP-EHH, which identifies regions where a long haplotype has reached fixation, or is close to fixation in one breed compared with other breeds [18]. We calculated the mean XP-EHH for all of the regions identified by the *S_i_* and *d_i_* tests. For the regions constructed from the top 1% of *d_i_* (6404 regions) and *S_i_* (7618 regions) statistics, mean XP-EHH was -0.94 and -1.13 respectively across all breeds compared with a genome average of zero. This difference is consistent across all 30 breeds and is highly significant (binomial test: *P*<10^−9^). This confirms that regions identified by the *S_i_* and *d_i_* tests are associated with unusually long haplotypes at or near fixation in the breeds under selection compared with other breeds.

### Functional categories

We analyzed genes closest to all singleton SNPs with high *F_ST_* for enrichment in gene ontology (GO) categories. The six most significantly overrepresented GO categories were all involved in development. 11 of the 22 genes were found in the “developmental processes category” (*P* = 0.00036) and tissue, system, organ, anatomical structure and multicellular organismal development were all significantly overrepresented (*P*<0.0007). These highly differentiated SNPs therefore highlight a number of regions involved in development that are likely to have been modified by artificial selection and contribute to the high diversity of dog breeds.

We next analyzed gene content of all of the regions constructed from the top 1% of *d_i_* and *S_i_* distributions that pass the marginal p-value <0.05 for each breed. We only considered regions containing a single gene, in order to enrich the analysis for true targets of selection, although this list is still expected to contain false positives. There were 119 *d_i_* regions and 272 *S_i_* regions containing one gene only (29 genes shared). We performed GO analysis using human genes with 1∶1 human-dog orthologous relationship. As longer genes are over-represented within long genomic segments containing only one gene, we compared these candidate selection genes to a background dataset with similar length (see Materials and Methods). A total of 40 GO categories were significantly enriched in the *S_i_* analysis and 6 in the *d_i_* analysis (Table 6). Developmental processes, central nervous system, organ development and pigmentation pathways are significantly enriched in *S_i_* regions whereas cell communication and signal transduction are the most represented in *d_i_* regions. These differences in enriched GO categories could potentially reflect differences in the form of selection detected by the two statistics.

**Table 6:** Enriched GO categories with 5 or more genes in *S_i_* and *d_i_* candidate selection regions.

GO ID	GO category	no. genes	enrichment	adjusted p-value
***S_i_*** ** regions**				
GO:0032501	multicellular organismal process	40	1.3	0.034
GO:0048869	cellular developmental process	22	1.5	0.032
GO:0030154	cell differentiation	21	1.6	0.021
GO:0048468	cell development	14	1.7	0.029
GO:0051239	regulation of multicellular organismal process	12	1.9	0.021
GO:0007186	G-protein coupled receptor protein signaling pathway	10	2.1	0.018
GO:0007507	heart development	6	2.9	0.016
GO:0030097	hemopoiesis	5	2.9	0.025
GO:0048534	hemopoietic or lymphoid organ development	5	2.7	0.033
GO:0048514	blood vessel morphogenesis	5	2.7	0.033
***d_i_*** ** regions**				
GO:0007154	cell communication	16	1.5	0.028
GO:0007165	signal transduction	15	1.6	0.027
GO:0007166	cell surface receptor linked signal transduction	8	2	0.04

A large number of genes detected by the *S_i_* analysis are significantly over-represented in several GO categories, which may reflect pleiotropic effects. A total of 23 of the genes belong to at least 10 enriched GO categories. As an example, one candidate selection gene the thyroid stimulating hormone receptor (TSHR) is involved in 25 enriched GO categories, including central nervous system and regulation of nucleotide biosynthetic process. This gene is suggested to have an essential role in photoperiod control of reproduction in vertebrates, in organ development and in metabolic regulation and has been recently been implicated as an important domestication gene in chicken [42].

The two larger biological processes over-represented by *d_i_* regions are ‘cell communication’ and ‘signal transduction’, which are represented by 16 and 15 genes, respectively. A region on chromosome 3 with strong statistical support contains the gene for insulin-like growth factor receptor1 (IGF1R), also detected by *S_i_* statistics. This is a strong candidate gene in relation to selection for growth, a phenotype that has been strongly selected in dog. Another example is ANGPT1, which plays roles in vascular development and angiogenesis and contributes to blood vessel maturation and stability. This gene has been identified in a set of positively-selected genes in human Tibetan populations for which selection may have occurred to allow for more efficient oxygen utilization [43]. The presence of TSHR and ANGPT1 in enriched GO categories may suggest that these pathways are commonly involved in recent adaptation.

### Fine-scale analysis of a region associated with multiple traits

**Figure 4:**
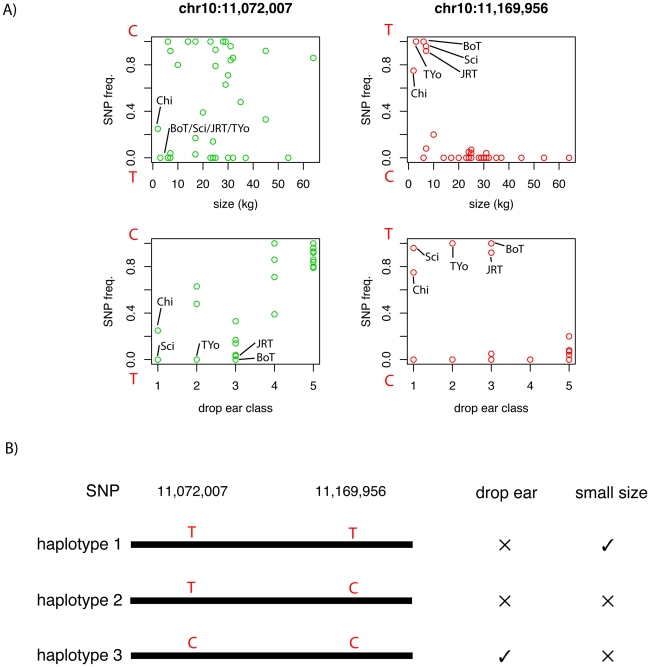
Variation in allele frequencies of the SNPs with the strongest association to drop ear (chr10:11,072,007) and body size (chr10:11,169,956). A) The frequency of these two SNPs in each breed is plotted against the classification of each breed according to body size and drop ear phenotype. The first SNP shows continuous variation in frequency between breeds, and correlates with drop ear class (1  =  pricked ear, 5  =  dropped ear). At the second SNP, one allele has very high frequency in some small breeds, but very low frequency in all other breeds. A set of small breeds with high minor allele frequency at this SNP are marked. B) The allele frequencies at these SNPs are consistent with the presence of three haplotypes, associated with different combinations of these traits.

The region containing the most highly differentiated SNPs identified by the single-SNP *F_ST_* analysis is 9.8 – 11.8 Mb on chromosome 10. Variation in this region was also found to correlate with multiple traits: drop ear, size and boldness. As boldness shows a strong correlation with the other traits, we focused on analyzing the contribution of variants in this region to drop ear and size. We first analyzed the variation in allele frequencies of the SNPs most associated with size and drop ear across breeds scored for these traits. Size was measured as the breed average in kg, and drop ear was scored on a scale of 1-5 (Figure 4A). The SNP most associated with ear type (chr10: 11,072,007) showed correlation with this trait, but little association with size. Allele frequencies display continuous variation between breeds. In contrast, the minor allele at the SNP most associated with size (chr10: 11,169,956) was not present in most breeds, but close to fixation in a subset of small breeds (Chihuahua, Yorkshire Terrier, Border Terrier, Schipperke, Jack Russell Terrier). All of these breeds were also fixed for the prick ear allele at the ear type SNP. Based on this analysis, we hypothesize that combinations of two alleles at these two SNP loci result in three main haplotypes affecting ear type and body size segregate among dogs (Figure 4B). The small size-pricked ear combination is present in the small (non-chondrodysplasic) breeds mentioned. All other breeds genotyped possess the large-prick or large size-drop ear haplotype, and the small size-drop ear combination is not observed in our dataset.

**Figure 5:**
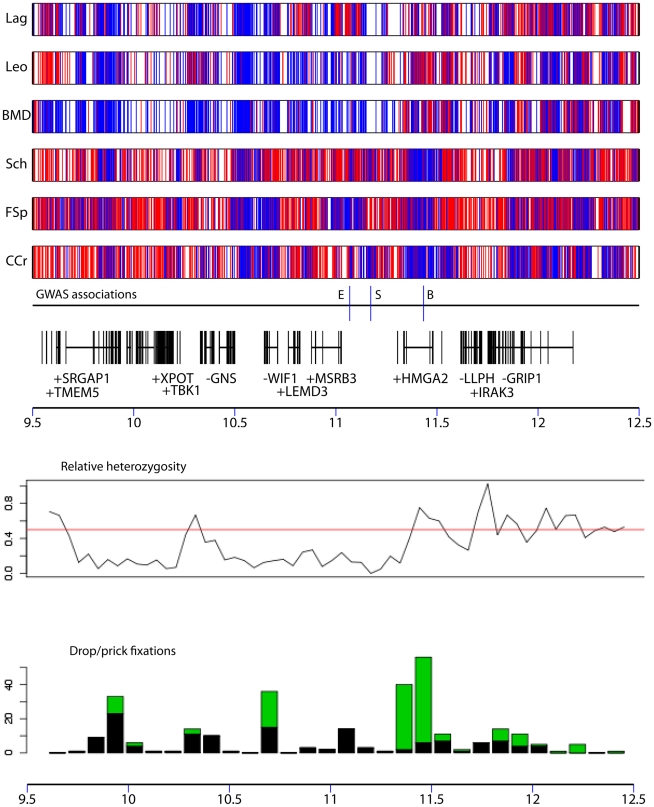
Patterns of polymorphism in a 3 Mb region where variation is associated with drop ear, body size, and boldness phenotypes. The top panel describes variation compared to the reference sequence in pools of 3 drop ear (Lagotto, Lag; Leonberger, Leo; Bernese Mountain Dog; BMD) and 3 pricked ear breeds (Schipperke, Sch; Finnish Spitz, FSp; Chinese Crested, CCr). Blue lines represent homozygous (fixed) differences from the reference sequence and red lines represent SNPs that are polymorphic in the breed pool. The positions of GWAS associations for drop ear (E) body size (S) and boldness (B) are shown. The positions of genes are also displayed (vertical bars correspond to exons). The second panel displays the levels of relative heterozygosity in all drop ear breeds compared with all prick ear breeds in 100 kb windows. The third panel shows the number of SNPs that are fixed for different alleles in drop and prick ear breeds. Green segments represent SNPs fixed for the reference allele in drop ear breeds, and black segments represent SNPs fixed for the reference allele in pricked ear breeds.

In order to identify variants potentially responsible for these traits, we comprehensively characterized variation in a genomic segment encompassing this region (chr10: 9.5 Mb – 12.5 Mb) using Roche NimbleGen hybrid capture and sequencing using an Illumina Genome Analyzer. We choose 3 breeds with the dropped ear phenotype (Lagotto Romagnolo, Leonberger, and Bernese Mountain Dog) and 3 with the pricked ear phenotype (Chinese Crested, Schipperke, and Finnish Spitz). Two of the pricked ear breeds are small, with breed average <6 kg: Chinese Crested and Schipperke. We sequenced each breed independently, using a pool of 5 dogs from each breed. On average 8 million reads per pool were produced, of which 48% mapped to the 3 Mb region on chr10. In total, 61% of this region was mapped by at least one read. In the 68% of the region defined as non-repetitive, reads mapped to 98% of bases, at an average coverage depth of 114x. By comparison with the reference sequence, we identified fixed differences and polymorphic sites within each breed. Differences in the pattern of polymorphism between dropped and pricked eared dogs are clearly apparent, and drop eared breeds exhibit a lower level of variation on average compared with prick eared dogs, which is mainly restricted to a ∼2 Mb region between 9.5 Mb and 11.5 Mb (Figure 5).

We next identified SNPs in this region that were completely fixed for different alleles in dropped and pricked eared breeds. These SNPs are distributed unevenly across the region, and a peak in the number of such fixed SNPs occurs around 11.3-11.5 Mb. In total 287 SNPs or small indels were completely fixed for different alleles in the drop ear compared with pricked ear breeds. Twenty-five of these SNPs reside in regions that show evidence for sequence conservation and are therefore candidates for being the causative mutation (Table S8). Of the 6 breeds, only Chinese Crested was completely fixed for the small size allele at chr10: 11,169,956 in our dataset. We therefore identified SNPs fixed for different alleles in Chinese Crested compared with all other breeds except Schipperke (this breed was excluded because it is small but was not completely fixed for the size-associated SNP from the GWAS). In total 297 SNPs or small indels were completely fixed for different alleles in these two groups. Of these, 17 were in conserved regions and are therefore candidates for affecting size (Table S9).

## Discussion

Here we present a comprehensive catalogue of genomic regions that are candidates for being affected by artificial selection in dogs using the densest panel of SNPs to date. We focus on two main types of variant: 1) common variants that affect variation in a trait in many breeds and 2) rare variants that have undergone selective sweeps in one or a few breeds. For the first category, we identify loci where variation correlates with morphological traits such as body size and tail curl, and behavioral traits such as sociability and boldness. We also identify several loci with evidence for a high degree of population differentiation between breeds, for which the connection with phenotypic traits in dogs is not known, but that are known to associate with traits such as pigmentation and body size. To identify loci in the second category, we searched for regions with reduced heterozygosity and high population differentiation, characteristic of selective sweeps. This analysis identified loci known to be associated with breed-defining characteristics such as chondrodysplasia, skin wrinkling, and furnishings. In addition, we identify several extended regions with reduced heterozygosity > 1 Mb consistent with recent selective sweeps in one or more breeds, including striking examples such as a region containing the FNDC3A and CYSLTR2 genes, and a region containing the MSTN (myostatin) gene that both bear the signal of selection in multiple breeds.

The candidate selection loci we identified are strongly enriched for genes involved in developmental and metabolic processes. In general, the GO terms we find to be significantly enriched are different from analyses of selection in natural populations, in which genes commonly targeted by positive selection include those involved in immunity and defense, olfaction and responses to external stimuli [36]. These results are consistent with the idea that artificial selection in domestic animals target different functional categories than natural selection. This result contrasts with that of Akey et al. [15] who found genes involved in immunity and defense to be overrepresented among their candidate selection regions.

Artificial selection on dog breeds coincided with breed creation bottlenecks leading to genetically distinct breeds fixed for novel traits [1], [3], [4]. Hence a large proportion of phenotypic and genetic variation is apportioned between but not within breeds. It is notable that 35% of polymorphic SNPs we analyzed are fixed or almost fixed for alternative alleles in two or more breeds. This is in sharp contrast to the differences between human populations, where only 78 near-fixed differences, that are all strong candidates for being under selection, were observed between four populations among 15 million SNPs identified using whole-genome resequencing [44]. The strong influence of genetic drift on genetic variation in dog breeds has also led to random fixation of long haplotypes and it is estimated that on average ∼25% of the genome lies within a homozygous block >100kb in an average breed. This suggests that functional genetic variation has also been affected by genetic drift. This background of fixation of haplotypes by drift makes it extremely difficult to distinguish the signal of a selective sweep from background variation, and they may often be indistinguishable.

We performed coalescent modeling using realistic estimates of recombination and demographic parameters in order to compare the length distributions of genomic segments identified by our analyses with those expected under neutrality. These simulations are by necessity an approximation of the actual evolutionary and demographic forces that shaped patterns of genetic variation in dog breeds. In particular, we do not model selection, which may reduce effective population size. Secondly, we assume a simplified demographic model, involving a single domestication bottleneck, and simultaneous breed creation. The true history of dog evolution is likely to be more complex than this, with some breeds showing closer relatedness than others. Nevertheless, long segments identified by the *S_i_* and *d_i_* that pass the 5% FDR cut off are strong candidates for selective sweeps, and contain a number of regions already associated with phenotypic traits.

Simulations indicate that large segments of reduced heterozygosity and elevated *F_ST_* are expected under neutrality but longer segments of reduced heterozygosity, particularly those longer than 1 Mb, are not expected to occur due to drift alone and hence are more likely to reflect selection. In general we expect segments of reduced heterozygosity to contain causative variants under selection, however, in some cases we observe large blocks of reduced heterozygosity that appear to be broken up into adjacent regions separated by more variable regions. This pattern may reflect heterogeneity in ancestral haplotypes, which makes it difficult to pinpoint the focus of selection. Smaller blocks of elevated *d_i_* often occur within extended regions of reduced heterozygosity. These probably reflect the fixation of variants that are otherwise rare in the dog population due to hitchhiking on the selected haplotype. However, most variants that are fixed by hitchhiking during a selective sweep are likely to be already common in the population, and therefore will not have a big effect on the *d_i_* value of a region. This leads to stochasticity in the *d_i_* statistic, which may explain the fact that even the longest *d_i_* segments still do not pass a 5% FDR. When even denser surveys of SNP variation (e.g. from whole genome sequencing) are available, a more promising approach could be to identify selective sweeps using reductions in heterozygosity, and identify potential causative variants within these sweeps by their elevated *F_ST_* (see e.g. [45]).

In addition to aiding in the dissection of the genetic components of phenotypic variation in dog breeds, we anticipate that our fine-scale map of genomic regions of extreme population differentiation and fixation of extended haplotypes will find utility for identification of disease causing variants. Firstly, regardless of whether they are caused by selection or drift, regions with reduced heterozygosity in a particular breed are problematic to interrogate with GWAS and may harbor disease-causing variants that are not tagged on a SNP array. Secondly, genetic variants responsible for breed characteristics may have pleiotropic effects that increase incidence of disease in that breed. Thirdly, disease-causing mutations may have risen in frequency in regions under selection by genetic hitchhiking on haplotypes bearing variants under artificial selection. These considerations suggest that our candidate selection regions warrant additional scrutiny in disease mapping studies. An example of the second effect has recently been highlighted in the Shar Pei breed, where strong artificial selection for genetic variants that likely affect expression of the HAS2 gene is responsible for both the characteristic wrinkled skin of the breed and an increased predisposition to periodic fever syndrome [16].

Our analysis of single-SNP *F_ST_* across breeds identified a number of extended genomic regions of extreme population differentiation between dog breeds, which harbor variants responsible for commonly varying traits between dog breeds. Genetic variation in some of these regions correlates with multiple traits that vary between dog breeds, in some cases including both morphological and behavioral differences. There are several possible reasons for these multiple associations. One possibility is that these regions harbor multiple variants that each has an effect on different traits. Alternatively the associations could be the result of single mutations with pleiotropic effects that affect multiple traits. It is also possible that traits may correlate with each other for other reasons. For example, there may have been coordinated selection for more than one trait in a subset of breeds, or a subset of breeds may share a trait simply by chance. We have comprehensively surveyed genetic variation in a region of extreme population differentiation on chromosome 10, where genetic variation correlates with body size, drop ears and boldness. As boldness shows strong correspondence with drop ears it is unclear whether this trait is affected by an independent variant in this region. A more detailed analyses of the allele frequencies of SNPs associated with body size and drop ears is consistent with a hypothesis that these traits are controlled by two linked SNPs, which in combination produce three observed haplotypes associated with distinct phenotypes. It is therefore possible that additional regions of extreme population differentiation also harbor multiple variants affecting different traits. Careful genetic dissection of each region is necessary to identify all functional variants and the traits they affect. As extensive LD is found in these regions, it is difficult to determine how many functional variants are present and their precise location. Such analysis would therefore be aided by the use of multiple breeds or populations with less extensive LD in order to narrow down the associated intervals.

In its most extreme form, a selective sweep is characterized by the rapid fixation of a new mutation under selection along with linked genetic variants (a hard sweep). However, less extreme selective episodes (soft sweeps), such as incomplete selective sweeps or selection on standing variation may also be common [46], [47]. It has been argued that polygenic adaptation, where subtle changes in allele frequencies occur at many loci, is the dominant form of phenotypic evolution in natural populations [48]. This type of evolution is likely when variation in a trait of interest is controlled by a large number of loci with small effect, which is now known to be the case with a number of highly heritable quantitative metabolic and morphological traits in humans. A long-term selection experiment in *Drosophila melanogaster* also uncovers evidence for this kind of adaptation [49]. Artificial selection in dogs appears to have caused genetic variants with much larger phenotypic effects to segregate at high frequencies, resulting in the simplification of the genetic architecture of phenotypic variation. In some cases, breed-defining characteristics such as chondrodysplasia, skin wrinkling and brachycephaly are likely to result from hard sweeps at breed creation. However, many variants with large phenotypic effects appear to show continuous variation between breeds that correlates with particular traits, including genetic variants that associate with body size in the IGF1 locus on chromosome 15 and with drop ear on chromosome 10, suggesting that selection by attenuation of allele frequencies is also common. Hence, although hard sweeps are likely to be a more common form of selection in domestic compared with wild species, it is likely that more minor changes in allele frequencies across many loci also contribute to phenotypic evolution.

The huge phenotypic diversity present in dogs raises the question as to whether levels of functional genetic variation in the ancestral dog population were elevated, adding to the raw material that artificial selection could act on. Relatively higher levels of replacement amino acid changes are found in dogs compared with wolves, possibly indicating a relaxation of selective constraint [50], [51]. There are also a large number of loci in the dog genome polymorphic for the active SINEC_Cf elements [52], which may also contribute to functional genetic variation, although it is not known whether functional variation due to these elements is increased in dogs compared with wolves. It has also been suggested (and disputed) that the dog genome has a high intrinsic mutation rate [53], [54]. There is also great interest in looking for “domestication genes” by identifying loci under selection in domestic species compared to wild ancestors. Investigation of these processes that occurred in the ancestral dog population requires detailed comparisons of patterns of genetic variation in dogs and wolves. As the majority (>98%) of SNPs on the CanineHD array were discovered by comparisons of dog breeds, they are biased against fixed differences between dogs and wolves and wolf-specific SNPs. Additional SNP discovery in wolves is therefore necessary to unravel the evolutionary processes involved in early dog domestication. Whole genome resequencing of both dogs and wolves will be important for a more detailed understanding of these processes.

It is likely that artificial selection in dogs (and other domestic animals) has led to the proliferation of mutations with large effects. This has contributed to the success of the dog as a model for genetic dissection of phenotypic traits. Such variants are likely to be maladaptive in the wild, and may also increase susceptibility to disease. Hence examining regions under selection in breeds may aid in identification of genetic risk factors affecting susceptibility to disease. Studying the extreme variation in forms produced by artificial selection also gives us a window into studying the effects of selection in natural populations, as first realized by Darwin [55]. Understanding the effects of selection on the genomes of domestic animals should give us insight into understanding its effects on nondomestic species, including our own.

## Materials and Methods

### Ethics statement

Blood samples were taken from dogs by trained veterinarians according to relevant national and international guidelines.

### SNP discovery

We scanned the existing list of 2.8 million high quality SNPs and identified 1,555 regions >40 kb (gaps) with no known SNPs in non-repetitive DNA (588 of these are on chromosome X). Gaps >100 kb were divided into a series of shorter ones resulting in a set of 2,375 genomic segments with no known SNPs of average length 50kb. We designed a Roche NimbleGen sequence capture array containing probes matching on average 2.1 kb within each segment, giving a total of 5 Mb. This array was used to enrich pools of DNA from Belgian Shepherds, Irish Wolfhounds, West Highland White Terrier, Shar-Pei and wolves. The samples were then sequenced using an Illumina Genome Analyzer and aligned to the CanFam2 dog reference sequence using MAQ. We identified 4,353 novel SNPs (973 on chromosome X). After updating the canine SNP map with these variants the number of gaps >40 kb was reduced to 714 (392 on chrX).

### Design of CanineHD array

We selected SNPs from initial list of 2.8 million augmented by the resequencing to be included in the Illumina CanineHD array. We selected SNPs by scanning the genome using non-overlapping windows of length 11,500 bp (this length was calculated to return the desired number of SNPs). Every SNP in each window was scored and ranked according to a number of different criteria in order to maximize quality, coverage of the genome and a number of other factors according to a scoring criteria (Table S10). The main criteria considered for each SNP were Illumina design score, presence on a list of SNPs known to be informative for studies of canid phylogeny and presence on lists of previous dog SNP arrays (Affymetrix and Illumina). SNPs in repetitive DNA or those that required two bead types on the Illumina array were disfavored. We also included 13 Y chromosome specific SNPs presented in ref [56]. The resultant list was analyzed to identify possible duplicates or incompatibilities between primers. The problematic SNPs were removed, and the final SNP list was edited manually to produce a list of 200k bead types by removing SNPs with the smallest distance to other SNPs.

### Genotyping

Genotyping was performed by Illumina Inc., USA (Gentrain dataset) and Centre National de Genotypage, France (LUPA dataset). All data is available at: http://dogs.genouest.org/SWEEP.dir/Supplemental.html.

### GWAS

For each trait we performed a GWAS with plink (http://pngu.mgh.harvard.edu/~purcell/plink/), using a breed permutation procedure to determine genome-wide significance implemented using a perl script. Each sample within a breed was first assigned a phenotype corresponding to the breed-specific value of a trait. Traits were either coded as dichotomous or quantitative depending on how they were measured (see below). An association study was performed for each trait followed by a permutation procedure, where the phenotypes of each breed were randomized, always assigning an identical phenotype value to each sample within the same breed. For each GWAS, 1000 permutations were performed, and the real significance values at each SNP were compared to the maximum permuted values across all SNPs in order to calculate genomewide significance.

We used the full LUPA dataset of 46 breeds to perform breed GWAS. The phenotypic values used are shown in Table S2. Personality traits and size were considered as quantitative traits. Other traits were considered dichotomous, and breeds were divided as follows (breed abbreviations in Table 1):

#### Furnishing association

4 breeds with furnishings (BoT, IrW, StP, TYo) compared with all other breeds. Drop ear association: drop ear breeds (Bgl, BMD, BrS, CKC, CoS, Dac, Dal, ECS, ESS, ESt, GoS, LMu, NFd, ShP, Wei) compared with pricked ear breeds (BeT, Chi, CWD, Elk, Eur, FSp, Gsh, GSl, Hus, Sam, Sar, Sci). Curly tail association: straight tail breed (BoT, DaC, Dal, EBT, EBD, Gry, LRe) compared with curly tail breeds (Elk, Eur, FSp, GSD, Mop, Sar, Sci, Scn, ShP, Hus, Sam, TYo). Boldness association: bold breeds (ASh, BeT, BMD, BoC, BoT, Box, Dal, Dob, Elk, GSh, Hus, JRT, Rtw, Sam, Sci, Scn, ShP, TYo) compared with non-bold breeds (Bgl, BrS, Chi, CKC, Dac, EBD, ECS, ESS, ESt, FcR, GoS, Gry, GRe, IrW, LRe, NFd, NSD, StP, Wei).

The rs numbers corresponding to SNPs mentioned in the text are listed in Table S11.

### Phasing and imputation

We phased the genotypes in the reduced dataset containing 471 dogs from breeds with 10 or more samples using fastPHASE [57] version 1 with the default parameters. We analyzed each breed and chromosome separately, dividing the X chromosome into the pseudo-autosomal region (PAR) and nonrecombining portion. Missing genotypes were imputed by the software, and we subsequently removed all SNPs that were not polymorphic or had less than a 100% call rate in all dog samples. In total, 19,176 invariant SNPs were removed: 14,309 on the autosomes and PAR, and 1027 on the nonrecombining X chromosome. An additional 3,840 SNPs were removed due to poor call rate. This dataset was used for subsequent selection scans and coalescent modeling analyses.

### Scans for selective sweeps using *S_i_* and *d_i_* statistics

The *S_i_* statistic is a measure of the proportion of SNPs that are variable in a region in a particular breed relative to all other breeds. We first divided the genome into 150 kb sliding windows, overlapping by 25 kb. Each window contained on average 10 SNPs; windows with less than 5 SNPs were not retained in the analysis. The same sliding window coordinates were used for the *S_i_* and *d_i_* analyses. Given a pair of breeds *i* and *j* and a given genomic window, we define relative heterozygosity as:
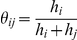
where *h_i_* is the number of polymorphic SNPs in breed *i* and *h_j_* is the number of polymorphic SNPs in breed *j* in a given genomic window.

*S_i_* for a given genomic window in breed *i* is then calculated as:
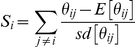
where *E[θ_ij_]* is the expected value of *θ_ij_*, calculated by comparing all of the SNPs between breed *i* and *j*, and *sd[θ_ij_]* is the standard deviation of all sliding windows. The *S_i_* statistic was calculated in this manner for all predefined 150 kb sliding windows across the genome, for all 30 breeds in the dataset. The *S_i_* statistic was calculated separately for the autosomal regions (including PAR) and the nonrecombining portion of the X chromosome, and was calculated in exactly the same way outlined above for the coalescent simulated data.

Using the same dataset, we calculated *F_ST_* for each pairwise breed combination. To identify regions with elevated *F_ST_* calculated the *d_i_* statistic for each SNP (Akey et al), which is a standardized measure of pairwise *F_ST_* values involving breed *i* and all other breeds:
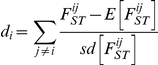
where E[*F_ST_^ij^*] and sd[*F_ST_^ij^*] represent respectively, the expected value and the standard deviation of *F_ST_* between breed *i* and *j* computed from all SNPs. For each breed, *d_i_* values were calculated for the 150 kb windows used for the *S_i_* analysis. We retained, for each breed, windows with an average *d_i_* within the top 1% of all *d_i_* values.

For each breed, we retained the top 1% of windows in each breed based on both *S_i_* and *d_i_* statistics. Overlapping windows were then combined to create a set of larger regions for each statistic. We applied this method to both the real and simulated data (see below) after which we compared the distribution of lengths. We then computed a marginal p-value for each region as the proportion of regions defined from the simulated dataset of the same breed that were longer. Finally we corrected the p-values using the Benjamini-Hochberg FDR method [33].

The UCSC graphical display of regions identified by the *S_i_* and *d_i_* statistics as well as *d_i_* values for all SNPs from the CanineHD array are available at the following URL:

http://dogs.genouest.org/SWEEP.dir/Supplemental.html

### Shared haplotype analysis

The aim of this analysis was to identify putative regions of Identity By Descent (IBD) within haplotypes inferred to be involved in selective sweeps in multiple breeds in order to narrow down the boundaries of putative sweep regions. This is based on the assumption that the selected variant was present on an ancestral haplotype prior to breed creation and is shared by multiple breeds. We first identified core regions that overlapped *S_i_* sweeps (at the 5% FDR) and were completely homozygous in each breed. Once these fixed regions were defined they were then grouped into clusters of overlapping physical locations between breeds. Where possible, we then identified the region of maximal overlap between all homozygous regions in all of the breeds in a cluster that had been fixed for identical haplotypes.

### Cross-population extended haplotype homozygosity (XP-EHH)

In order to calculate XP-EHH for SNPs in our dataset, we first removed SNPs with a minor allele frequency < 5% in the entire dataset. We calculated the EHH statistic between all SNP pairs across all breeds in the whole dataset. We retained SNP pairs with EHH between 0.03 and 0.05 for the XP-EHH analysis. We calculated normalized log XP-EHH scores between these SNP pairs from iHS scores as described by [18]. However, instead of comparing iHS score between pairs of populations, we compared iHS scores in a given breed and SNP pair to the average of iHS scores in all other breeds. The normalization step was performed for each chromosome in each breed separately. In order to confirm the presence of extended haplotypes in putative sweep regions, we averaged XP-EHH scores across these regions in each breed compared to the genomewide average.

### Coalescent simulations

We performed whole genome simulations under a realistic demographic model, using variable regional recombination rates as inferred from the original data. The simulation process consisted of three main steps: (1) recombination rate inference, (2) breed bottleneck modeling and (3) main simulations.

#### Recombination rate inference

We used interval in the LDhat package [58] to estimate the total population scaled recombination rate (ρ = 4Ner) of each dog chromosome, as well as regional recombination rate variation across all chromosomes. For each chromosome, we randomly chose 100 haplotypes from the original data as input for interval. We split the input data into consecutive blocks of 2000 SNPs, each overlapping the previous block by 200 SNPs. Recombination rate estimates from individual blocks were then concatenated to get chromosome wide rate estimates. Interval was provided a look up table downloaded from http://www.stats.ox.ac.uk/~mcvean/LDhat/instructions.html,which assumes a population mutation rate of 0.01. The PAR was analyzed separately from rest of the X-chromosome. To assess the general performance of interval on dog data, we averaged the regional rate estimates obtained here across 5 Mb windows to make it compatible with a previously published, coarse, linkage map [59]. The concordance between the population genetic map and the linkage map is good (Axelsson et al, unpublished). To convert the population scaled recombination rate estimated for the domesticated dog into breed specific rates we first estimated the effective population size of the domesticated dog (Ne_dog Autosomes_  =  7752) by comparing the autosomal part of the population genetic map generated here, with that of the linkage map [59]. Then to take a potential bias in reproductive success between males and females into account we used the same approach to estimate the effective population size using only the X-chromosome (Ne_dog X_ = 9134).

#### Modeling breed bottlenecks

We built on previous dog demographic modeling efforts in setting up a simple simulation scheme to estimate the strength of bottlenecks at breed formation for each breed in our dataset. Our model thus assumed an effective population size of the ancient wolf population (Ne_wolf_) of 22600 [60]. It furthermore assumed that dog domestication occurred 5000 generations ago, accompanied by an instantaneous decrease in population size to 5560Ne_dog_ and that breed formation took place 100 generations ago [60]. The mutation rate was set to 10^−8^ [1] and the generation time was assumed to be 3 years. We then used MaCS [61] to simulate genome wide replicas of our dataset according to the model described above, for 59 breed bottle neck sizes, ranging from 0.001Ne_wolf_ - 0.03Ne_wolf_ (this corresponds to an increment in bottle neck size of 0.0005Ne_wolf_ for every new simulation). We repeated these simulations for each of the sample sizes represented in the original data (ranging from 10 to 52 haplotypes), in total rendering 767 simulated datasets. All simulations were run using regional recombination rates as inferred in the real data. We also corrected simulated data for ascertainment bias in the original data by providing MaCS with allele frequency distributions from the original data. Next, we estimated LD decay, measured as r^2^, for markers separated up to a maximum of 500 kb, in the original, as well as all simulated datasets. We subsequently used least squares to fit LD decay curves of real and simulated datasets. The best fitting simulation provided an estimation of bottleneck size for each breed individually.

#### Main simulation

By implementing the estimated breed bottleneck sizes in the model described above we were then able to simulate a single complete dataset including all breeds in the original dataset. As before, regional recombination rates were inferred from the original data, and ascertainment bias in the original data was corrected for in the simulated data. Finally, we thinned the simulated dataset to match the marker density of the real dataset (one marker every 13,046 bp). The PAR and X-specific parts of the X-chromosome were simulated separately. For the X-specific simulations we adjusted all population sizes according to the difference between Ne_dog Autosomes_ and Ne_dog X_.

### Functional analysis of gene categories

We selected human orthologs with a 1∶1 human-dog orthologous relationship to perform GO analyses. Biomart version 0.8 (Ensembl v.62) was used to collect orthologous human protein-coding genes. WebGestalt [62], a web-based gene set analysis toolkit, was used to retrieve GO terms associated with human ensembl gene stable IDs. A hypergeometric test computed the statistical significance of over-representations of GO terms that were compared to a background list of genes selected to control for possible gene length bias as observed in the selected gene set. The background set was composed of human genes selected using biomart with 1∶1 human-dog orthologous relationship, longer than 100 kb and with a mean size of 230 kb, similar to the tested set. GO biological processes that were significantly over-represented (p<0.05) were considered.

### Resequencing of a candidate selection region on chromosome 10

We selected a 3 Mb region on chromosome 10 (9.5-12-5 Mb) for resequencing in 6 breeds. We first prepared pools of DNA containing 5 samples from each of the breeds (Chinese Crested, Lagotto, Schipperke, Finnish Spitz, Leonberger and Bernese Mountain Dog). We next performed sequence capture using a Roche NimbleGen array containing probes designed to hybridize to this region. This was followed by sequencing using the Illumina Genome Analyzer. Reads were mapped to the dog genome reference sequence using bwa (http://bio-bwa.sourceforge.net/) followed by SNP calling using samtools (http://samtools.sourceforge.net/). Mapping and SNP calling was done independently for each breed and custom scripts were used to identify SNPs with certain patterns of segregation. SNPs in conserved elements were identified relative to those defined by the dog genome analysis based on human-dog-mouse-rat alignments, and on identification of phastcons elements within mammals based on alignments of 44 vertebrates, converted from human to dog coordinates by LiftOver (http://genome.ucsc.edu/).

## Supporting Information

Figure S1 Coverage of HD array. Number of SNPs in 100 kb windows across the genome contained in the Illumina CanineHD array and the Affymetrix V2 Canine array on A) autosomes and B) X chromosome. For autosomes, there is an average of 9 SNPs per 100 kb window on the HD array and only 5% of windows do not contain SNPs, whereas the majority (>25%) of 100 kb windows do not contain SNPs on the Affymetrix array. For the X chromosome, >75% of windows do not contain SNPs on the Affymetrix array, whereas <5% of windows do not contain SNPs on the HD array.
(PDF)

Figure S2 Decay of linkage disequilibrium in real versus simulated data. Decay of linkage disequilibrium (LD decay) across the autosomes of all breeds included in this study (solid lines) compared with that of simulated datasets (circles). Wolf is included as comparison. LD decay was calculated as r^2^ for markers separated by at most 400kb and averaged across bins of 10kb. Simulation were run in MaCS [61] with the following general model; ancient Wolf Ne: 22600 [60], ancient domesticated dog Ne: 5650 [60], dog domestication 5000 generations ago [60], dog breed formation 100 generations ago [60], mutation rate: 1×10^−8^ per site per generation [1], generation time: 3 years. Each breed was then assigned (1) a specific breed bottleneck size (determined by simulation, see Table S1) from the following range: 0.001-0.03 x (ancient Wolf N_e_) as well as (2) a sample size to match that of the real dataset (10-52 haplotypes). Furthermore, recombination rates were allowed to vary locally as inferred in real data using LDhat [58]. We corrected for ascertainment bias by supplying MaCS allele frequencies from the real dataset.
(PDF)

Figure S3 Allele frequency of 3 SNPs strongly associated with body size in the dataset across breeds plotted against body size. Data on body size is presented in Table S3.
(PDF)

Figure S4 Signal of association with curly tail of chr1: 95-98 Mb. The y-axis shows the raw p-value and genes in the region are shown below the graph.
(PDF)

Figure S5 Signal of association between drop ear, boldness and size in the region chr10:9.5-12.5 Mb. The y-axis show the raw p-value for each association, and genes in the region are display beneath the graph.
(PDF)

Figure S6 Signal of association with sociability on chrX: 102-110 Mb. The y-axis shows the raw p-value and genes in the region are shown below the graph.
(PDF)

Figure S7 Map of extended segments in the dog genome of significantly reduced *S_i_* that pass the 5% FDR cut-off. Each region is color coded according to the breed in which the significant reduction is observed. The breed codes are shown in Table 1.
(PDF)

Figure S8 Examples of *S_i_* and *d_i_* statistics in top 10 longest *S_i_* regions. Variation in these statistics is shown independently in genomic segments encompassing each region.
(PDF)

Table S1 Total samples in dataset.
(DOCX)

Table S2 Breed bottleneck sizes used in simulations.
(DOCX)

Table S3 Phenotypes used in across-breed GWAs.
(DOCX)

Table S4 Single-SNP *F_ST_*. SNPs with minor allele frequency >0.15 and *F_ST_*>0.55. Nearby SNPs <500kb are clustered into regions.
(DOCX)

Table S5 (XLSX)

Table S6 (XLS)

Table S7 (XLS)

Table S8 (DOCX)

Table S9 (DOCX)

Table S10 (DOCX)

Table S11 (XLSX)
