# Supplementary figures and images for: Identification of Genomic Regions Associated with Phenotypic Variation between Dog Breeds using Selection Mapping
Source: PLoS Genet. 2011 Oct 13;7(10):e1002316. doi: 10.1371/journal.pgen.1002316 (PMC3192833; doi:10.1371/journal.pgen.1002316)

A)

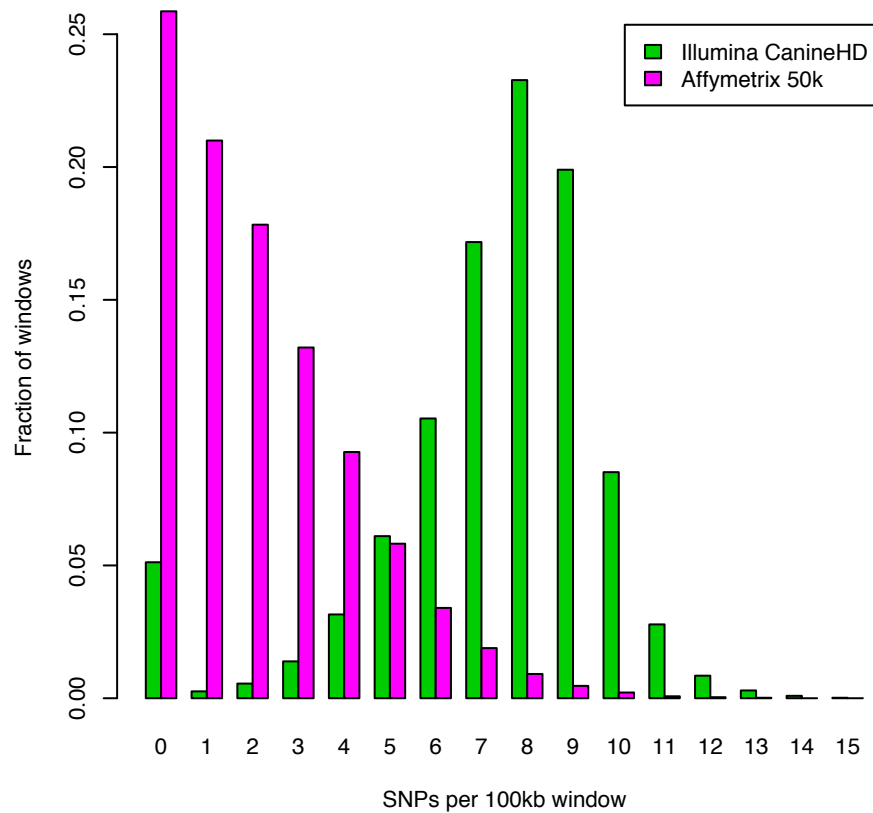

B)

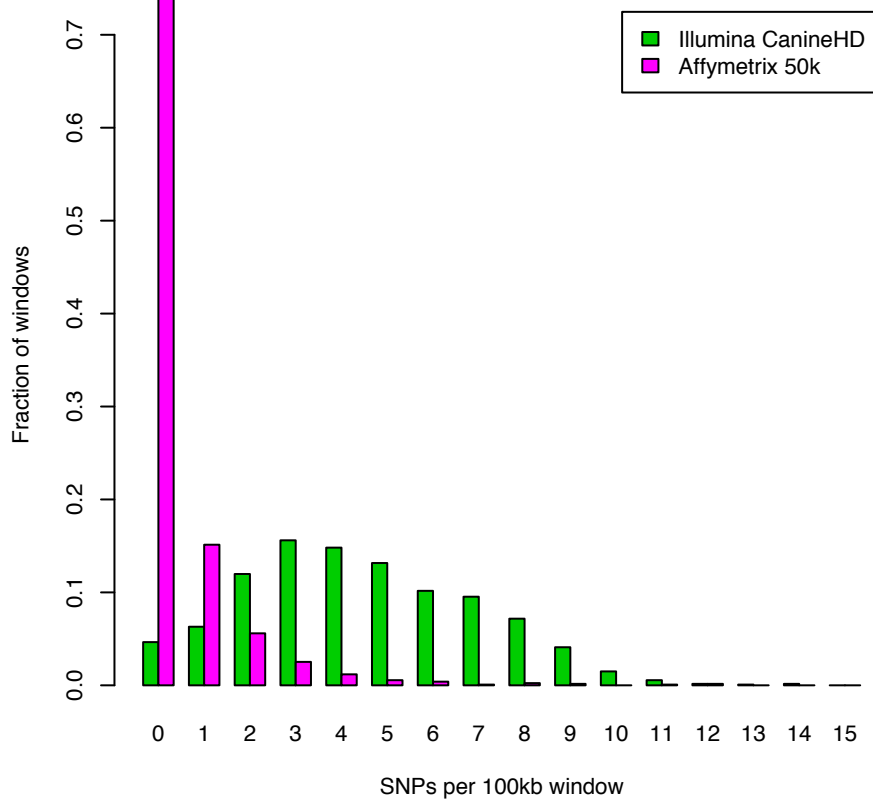

Supplement: Figure S1 — Coverage of HD array. Number of SNPs in 100 kb windows across the genome contained in the Illumina CanineHD array and the Affymetrix V2 Canine array on A) autosomes and B) X chromosome. For autosomes, there is an average of 9 SNPs per 100 kb window on the HD array and only 5% of windows do not contain SNPs, whereas the majority (>25%) of 100 kb windows do not contain SNPs on the Affymetrix array. For the X chromosome, >75% of windows do not contain SNPs on the Affymetrix array, whereas <5% of windows do not contain SNPs on the HD array. (PDF) [file pgen.1002316.s001.pdf]

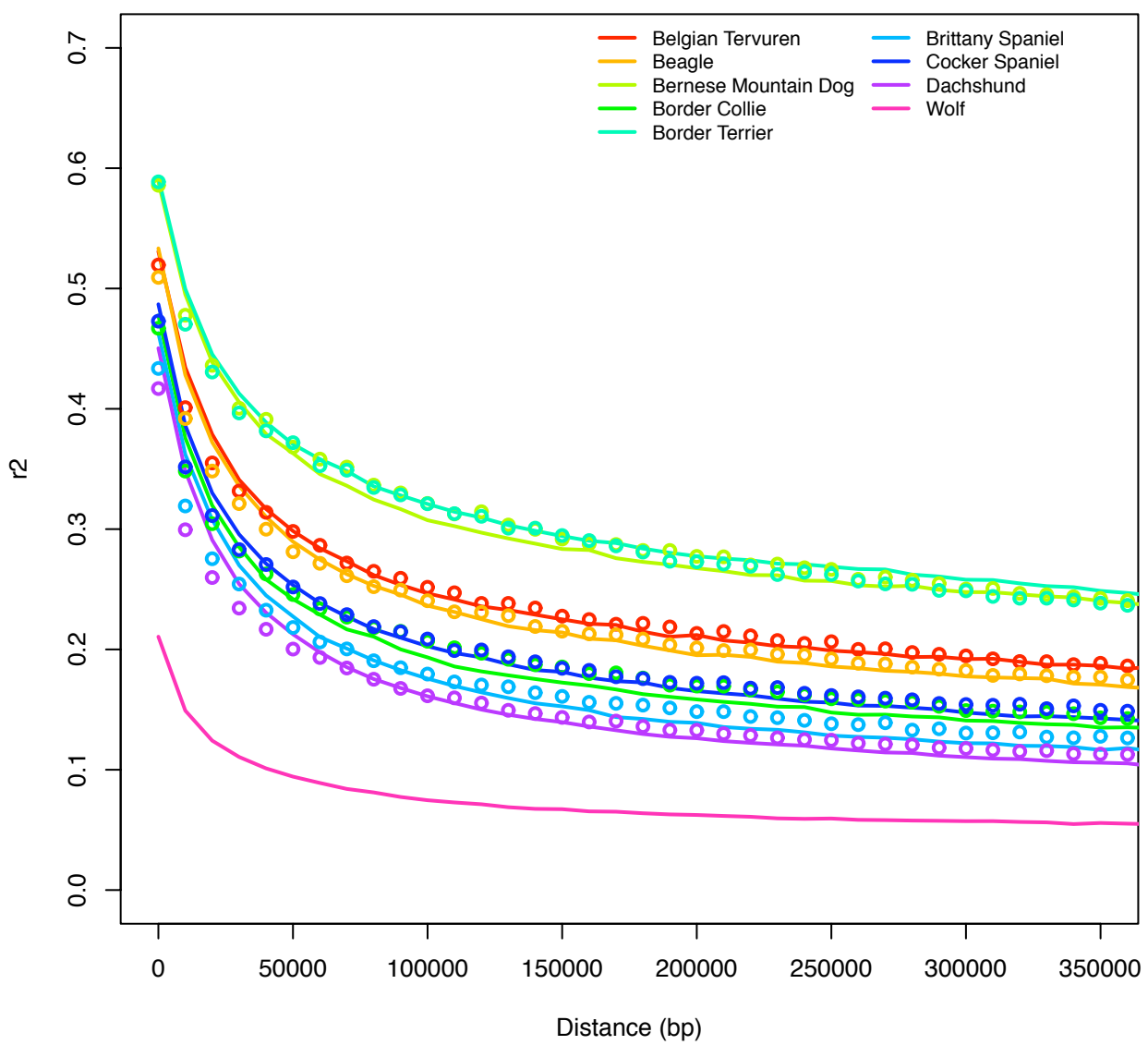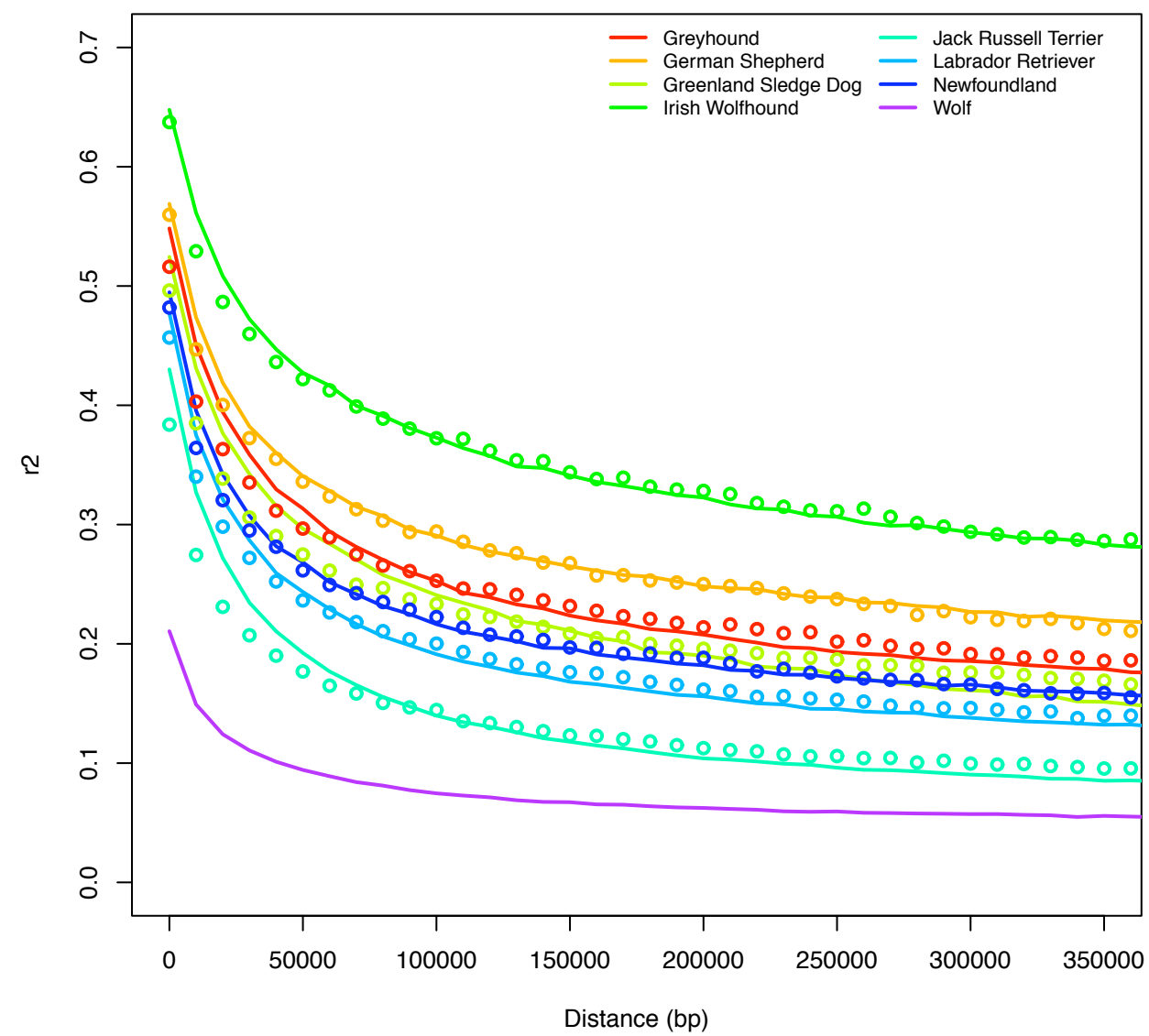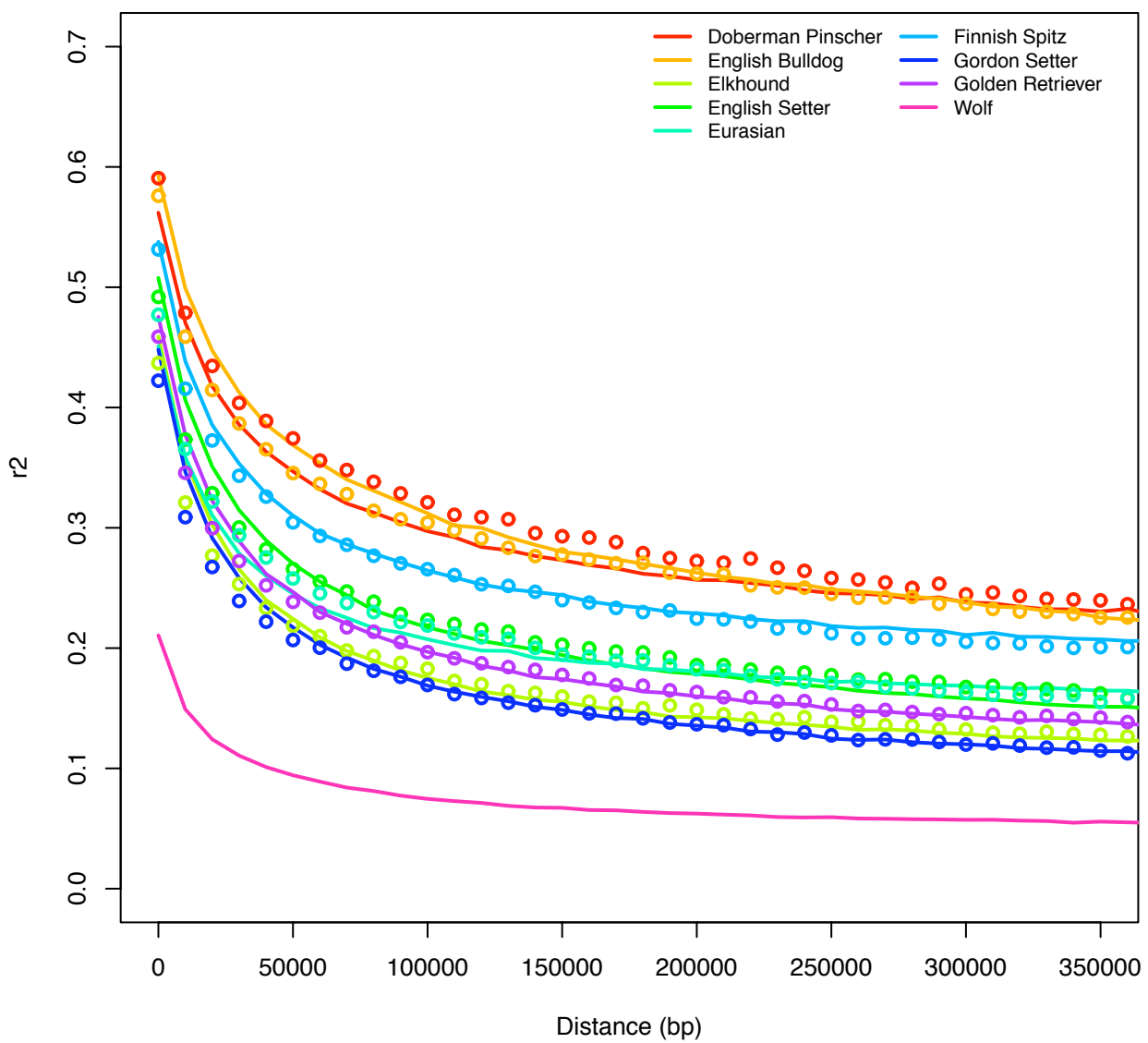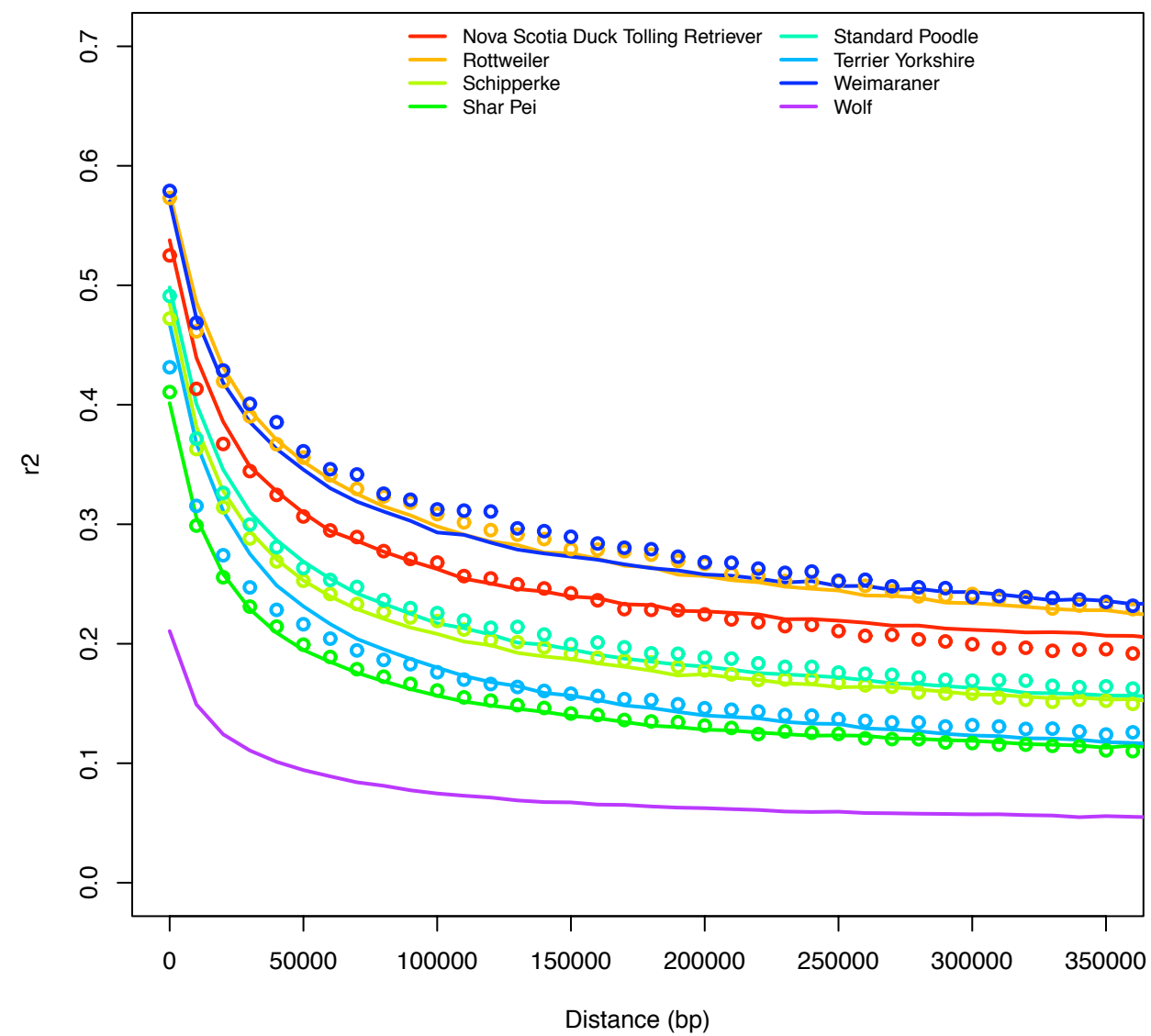

Supplement: Figure S2 — Decay of linkage disequilibrium in real versus simulated data. Decay of linkage disequilibrium (LD decay) across the autosomes of all breeds included in this study (solid lines) compared with that of simulated datasets (circles). Wolf is included as comparison. LD decay was calculated as r2 for markers separated by at most 400kb and averaged across bins of 10kb. Simulation were run in MaCS [61] with the following general model; ancient Wolf Ne: 22600 [60], ancient domesticated dog Ne: 5650 [60], dog domestication 5000 generations ago [60], dog breed formation 100 generations ago [60], mutation rate: 1×10^−8^ per site per generation [1], generation time: 3 years. Each breed was then assigned (1) a specific breed bottleneck size (determined by simulation, see Table S1) from the following range: 0.001-0.03 x (ancient Wolf Ne) as well as (2) a sample size to match that of the real dataset (10-52 haplotypes). Furthermore, recombination rates were allowed to vary locally as inferred in real data using LDhat [58]. We corrected for ascertainment bias by supplying MaCS allele frequencies from the real dataset. (PDF) [file pgen.1002316.s002.pdf]

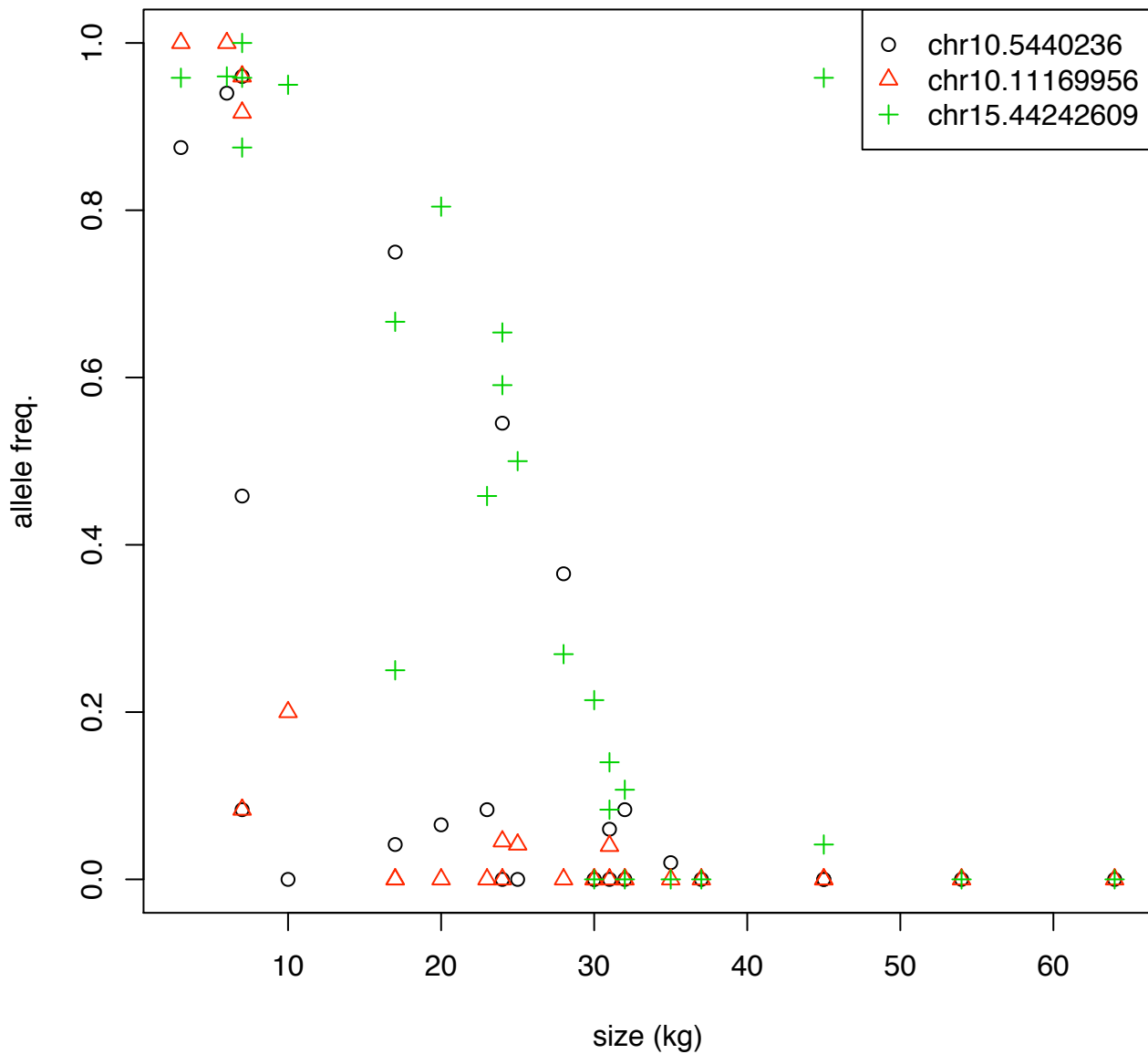

Supplement: Figure S3 — Allele frequency of 3 SNPs strongly associated with body size in the dataset across breeds plotted against body size. Data on body size is presented in Table S3. (PDF) [file pgen.1002316.s003.pdf]

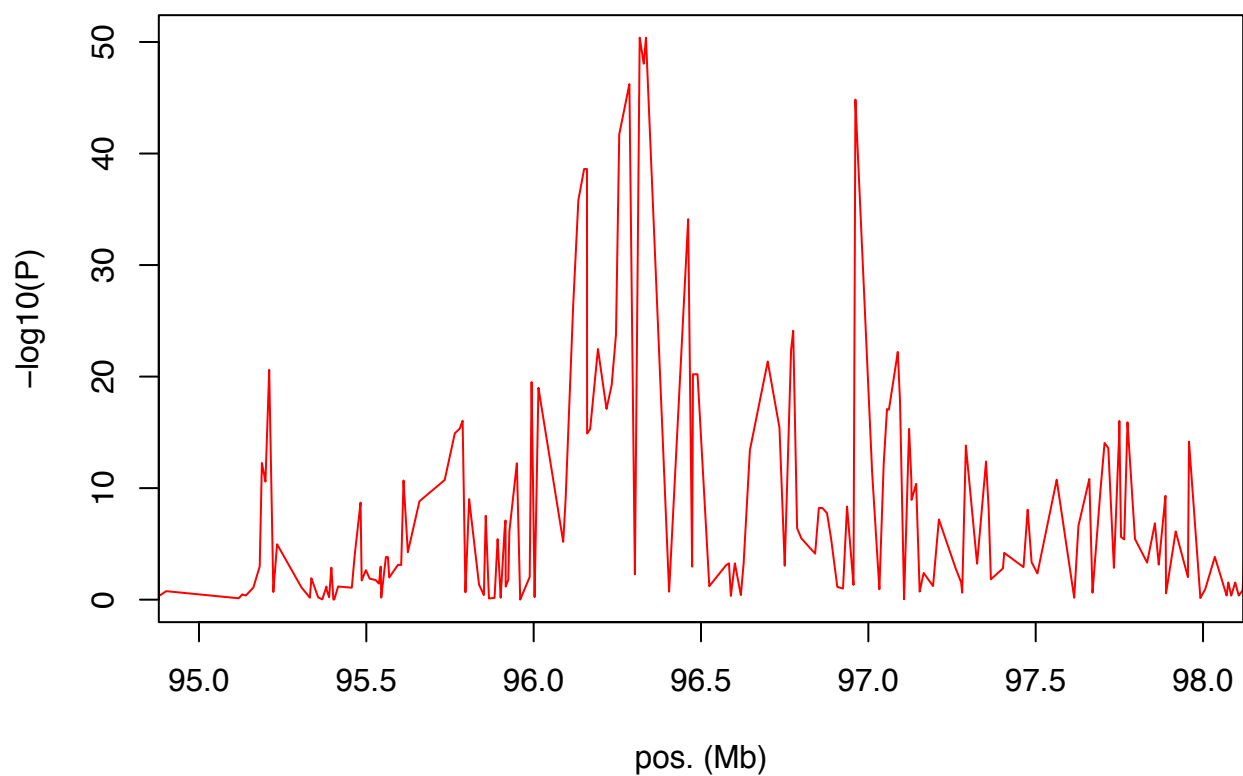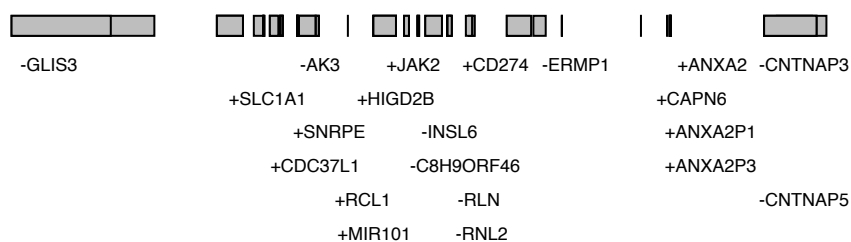

Supplement: Figure S4 — Signal of association with curly tail of chr1: 95-98 Mb. The y-axis shows the raw p-value and genes in the region are shown below the graph. (PDF) [file pgen.1002316.s004.pdf]

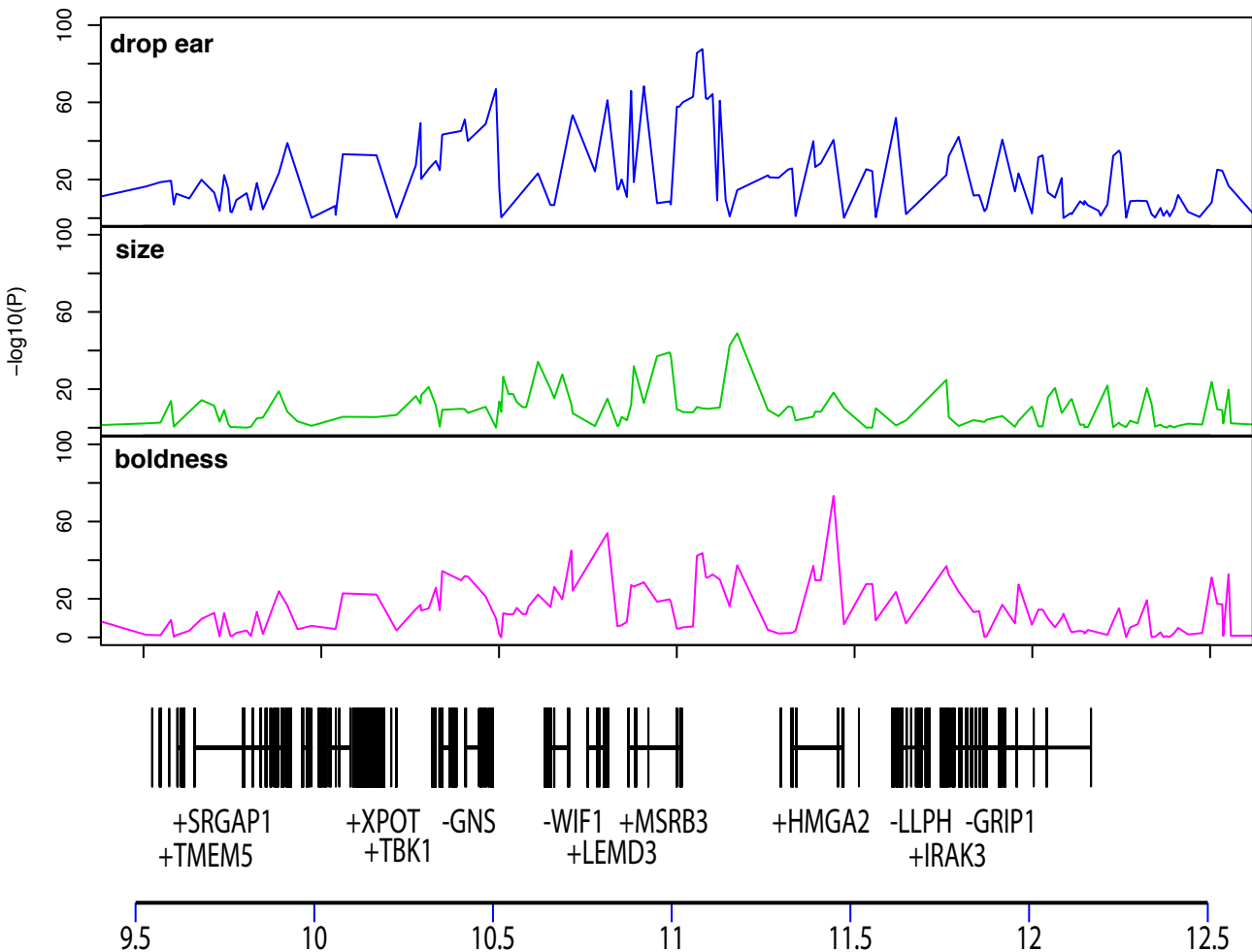

Supplement: Figure S5 — Signal of association between drop ear, boldness and size in the region chr10:9.5-12.5 Mb. The y-axis show the raw p-value for each association, and genes in the region are display beneath the graph. (PDF) [file pgen.1002316.s005.pdf]

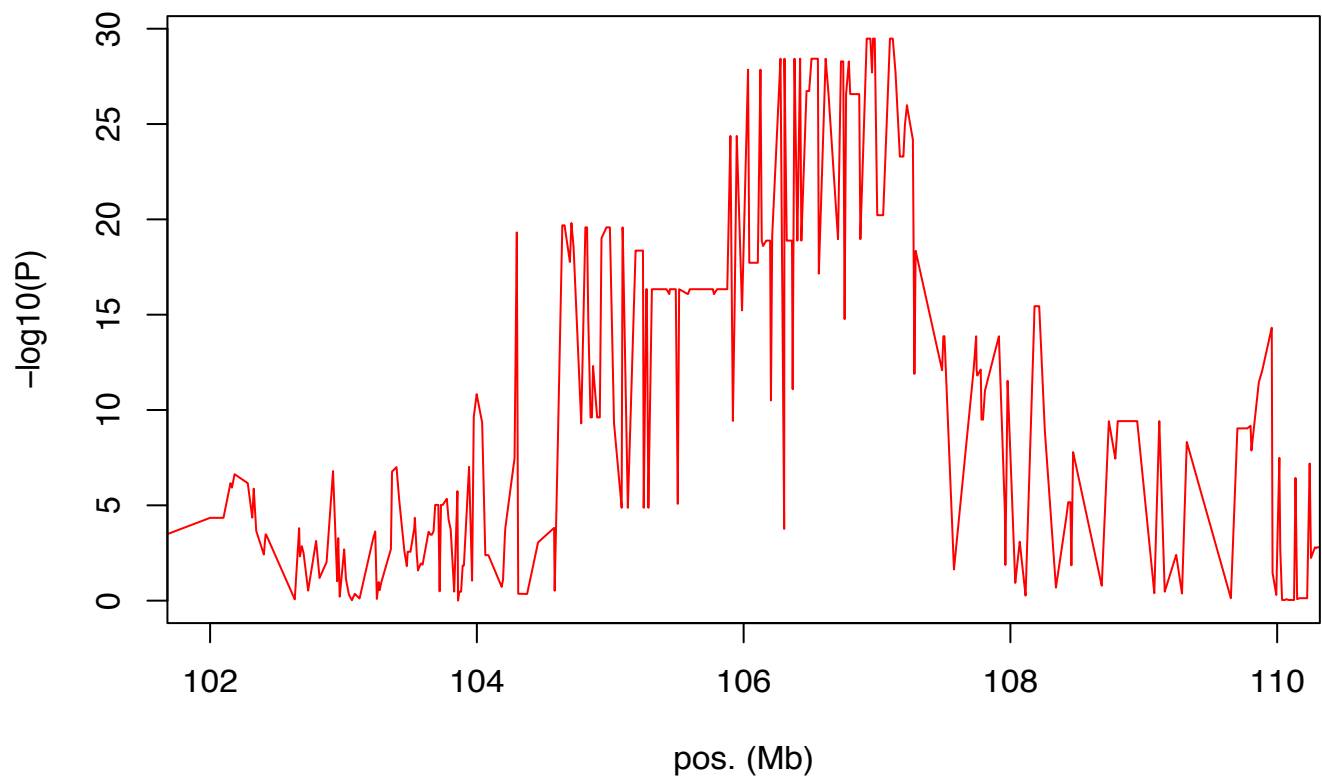

Supplement: Figure S6 — Signal of association with sociability on chrX: 102-110 Mb. The y-axis shows the raw p-value and genes in the region are shown below the graph. (PDF) [file pgen.1002316.s006.pdf]

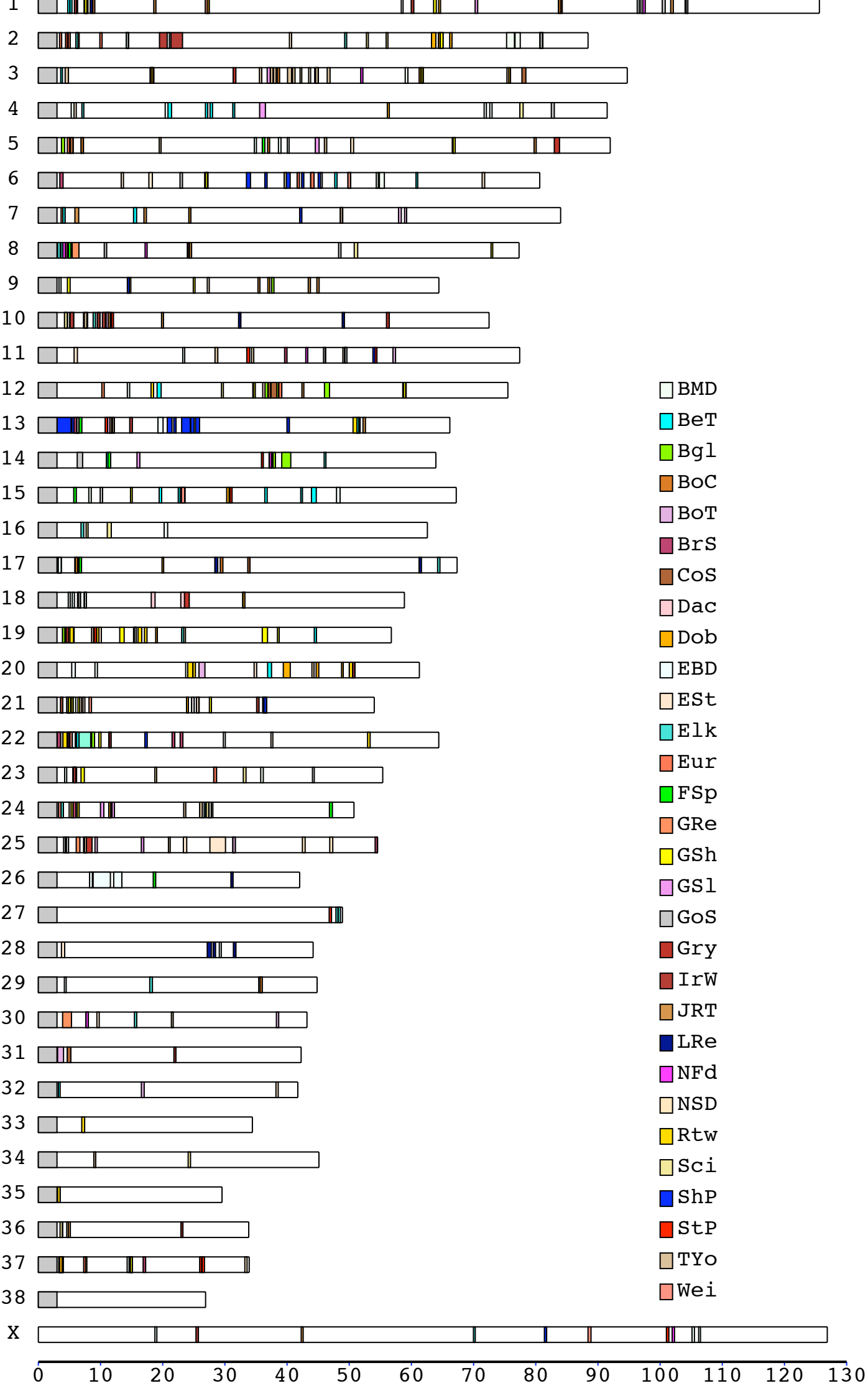

Supplement: Figure S7 — Map of extended segments in the dog genome of significantly reduced Si that pass the 5% FDR cut-off. Each region is color coded according to the breed in which the significant reduction is observed. The breed codes are shown in Table 1. (PDF) [file pgen.1002316.s007.pdf]
