# Supplementary material for: Identification of Genomic Regions Associated with Phenotypic Variation between Dog Breeds using Selection Mapping
Source: PLoS Genet. 2011 Oct 13;7(10):e1002316. doi: 10.1371/journal.pgen.1002316 (PMC3192833; doi:10.1371/journal.pgen.1002316)
Supplement: Figure S8 — Examples of Si and di statistics in top 10 longest Si regions. Variation in these statistics is shown independently in genomic segments encompassing each region. (PDF) [file pgen.1002316.s008.pdf]

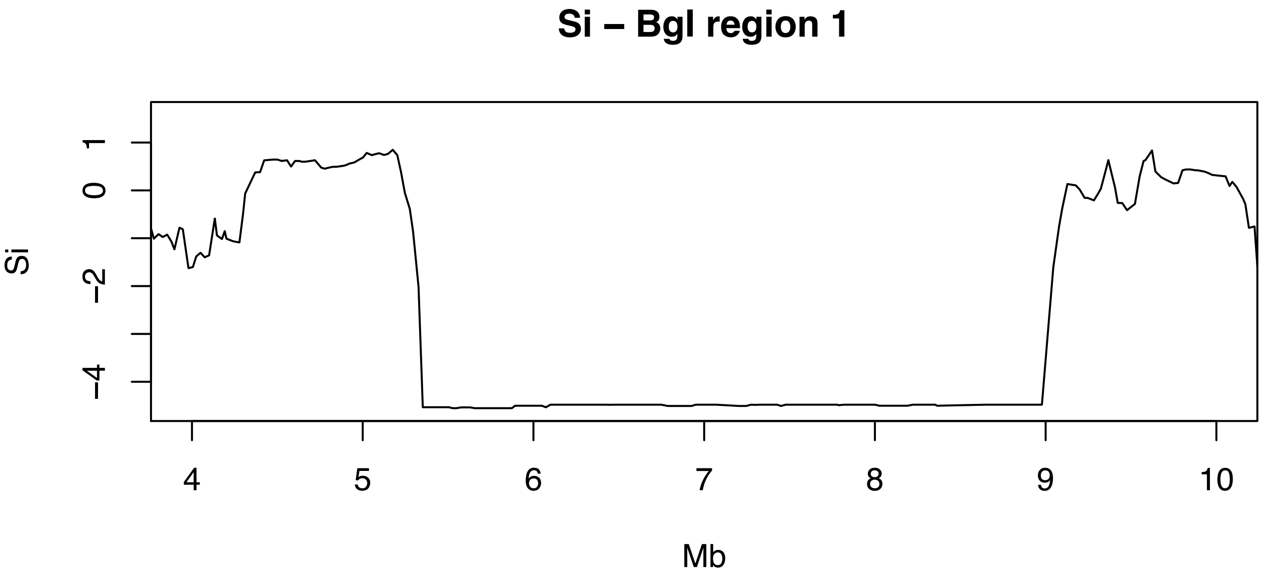

Si – Bgl region 1
Si
1
0
−2
−4
4
5
6
7
8
9
10
Mb

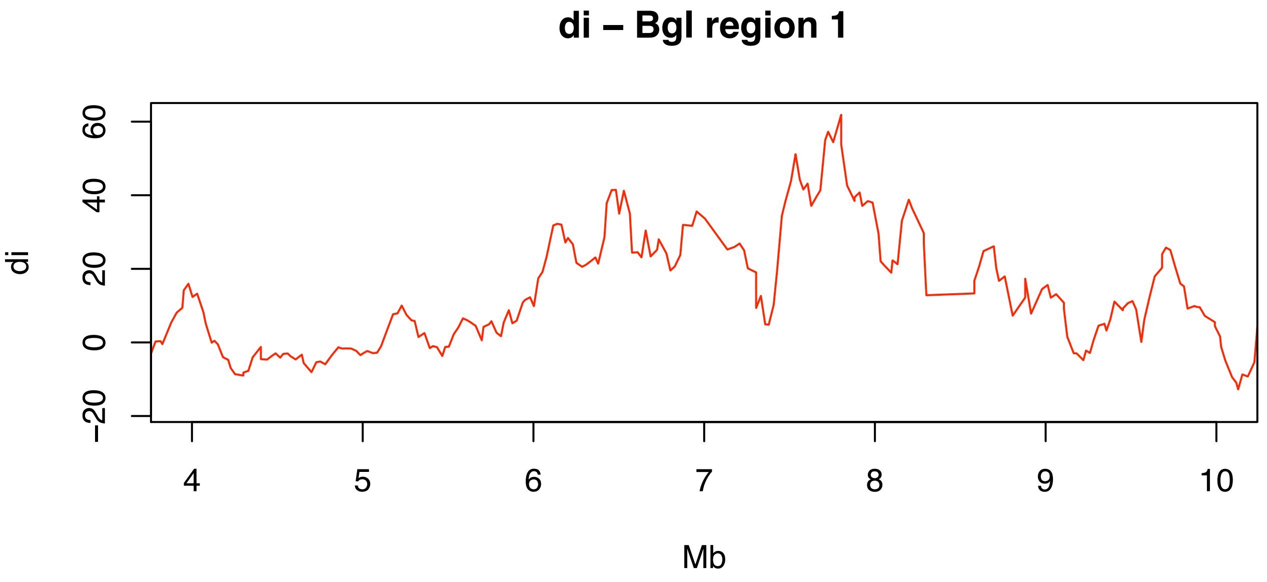

di – Bgl region 1
di
60
40
20
0
−20
4
5
6
7
8
9
10
Mb

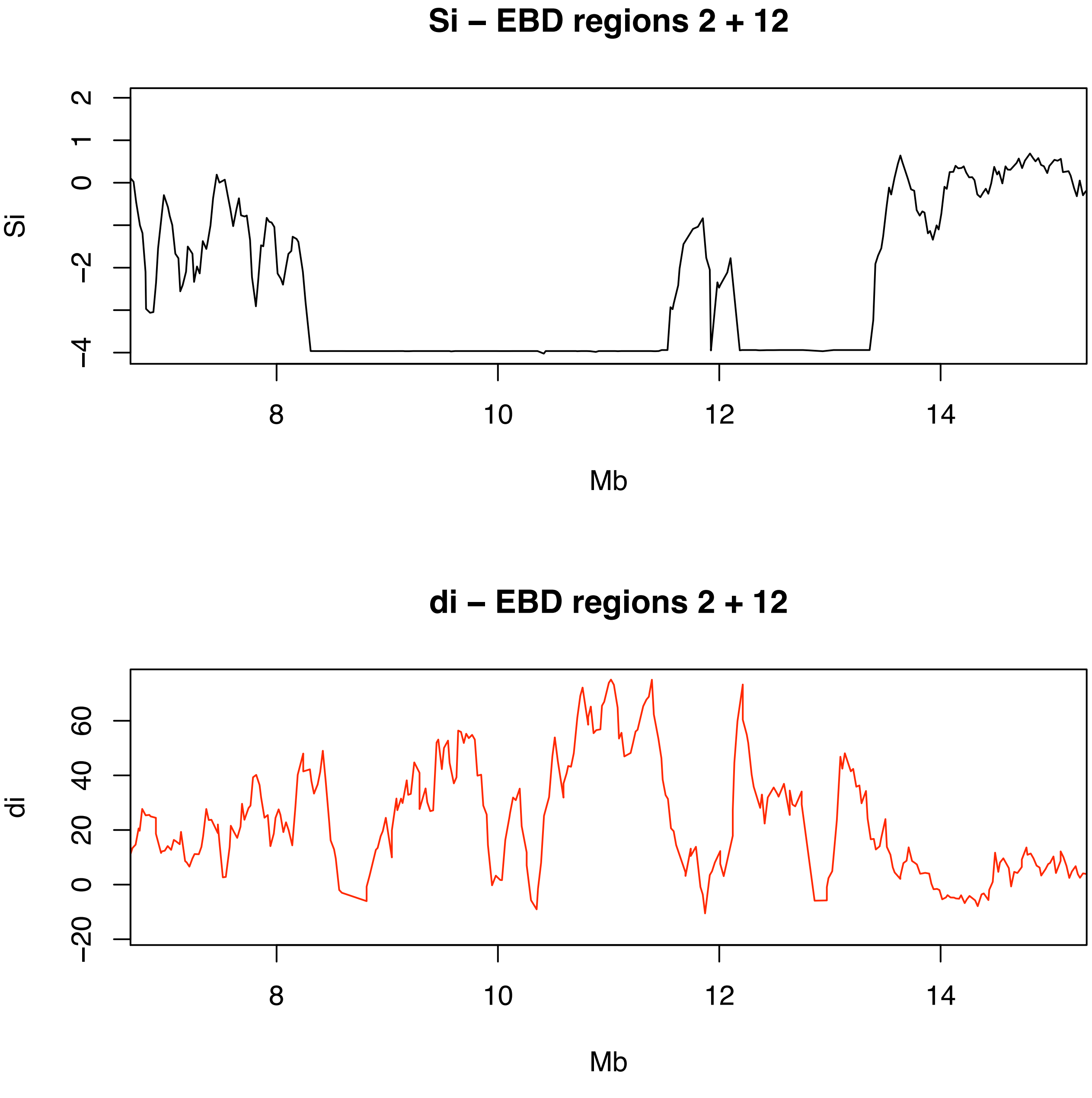

Si – EBD regions 2 + 12
Si
2
1
0
−2
−4
8
10
12
14
Mb
di – EBD regions 2 + 12
di
60
40
20
0
−20
8
10
12
14
Mb

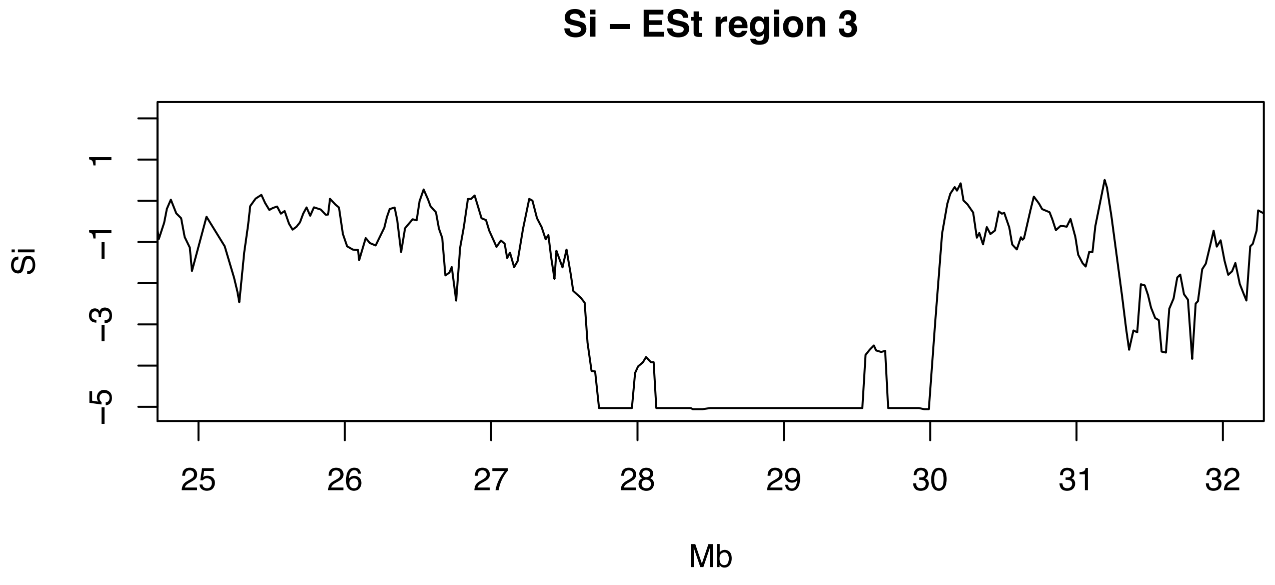
Si – ESt region 3
Si
1
−1
−3
−5
25
26
27
28
29
30
31
32
Mb

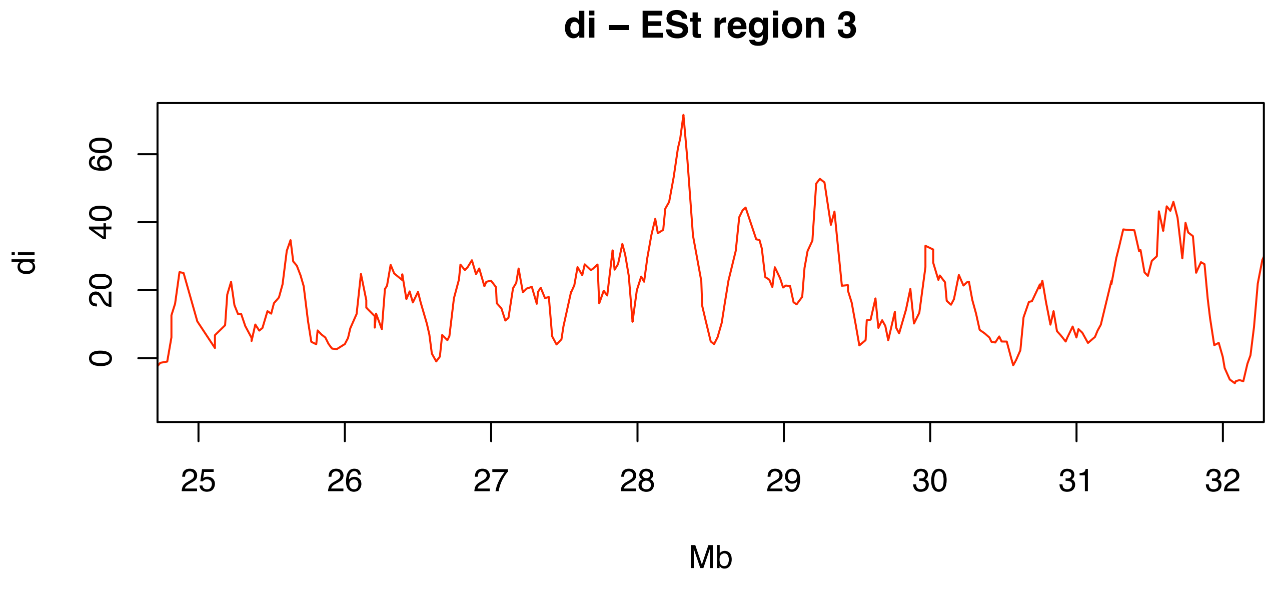
di – ESt region 3
di
0
20
40
60
25
26
27
28
29
30
31
32
Mb

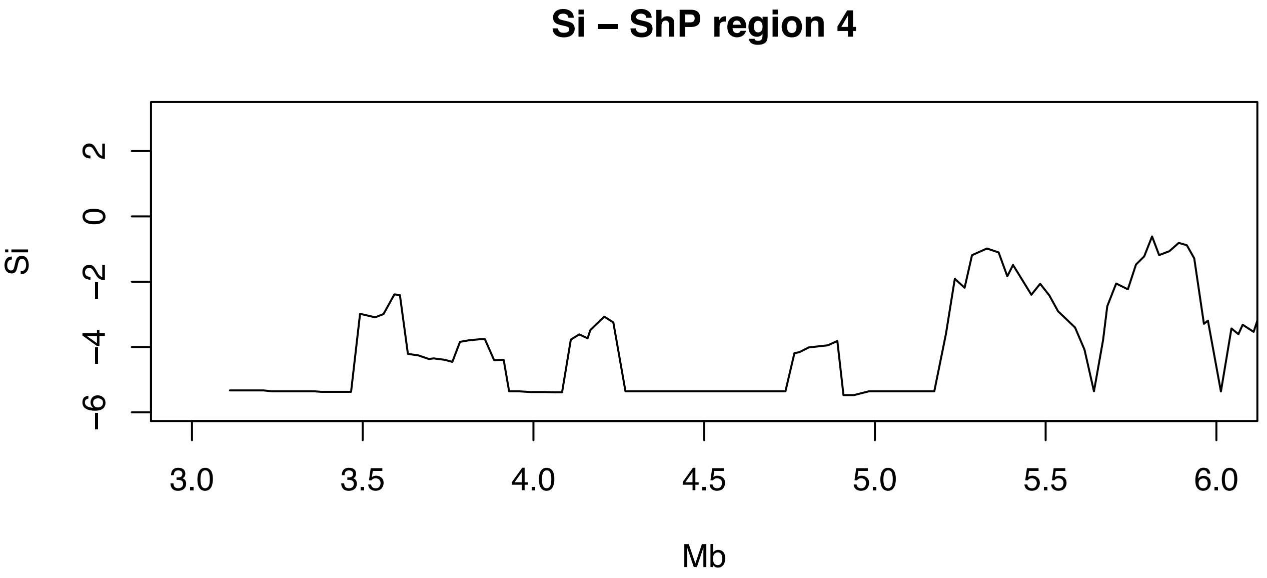
Si – ShP region 4
Si
2
0
−2
−4
−6
3.0
3.5
4.0
4.5
5.0
5.5
6.0
Mb

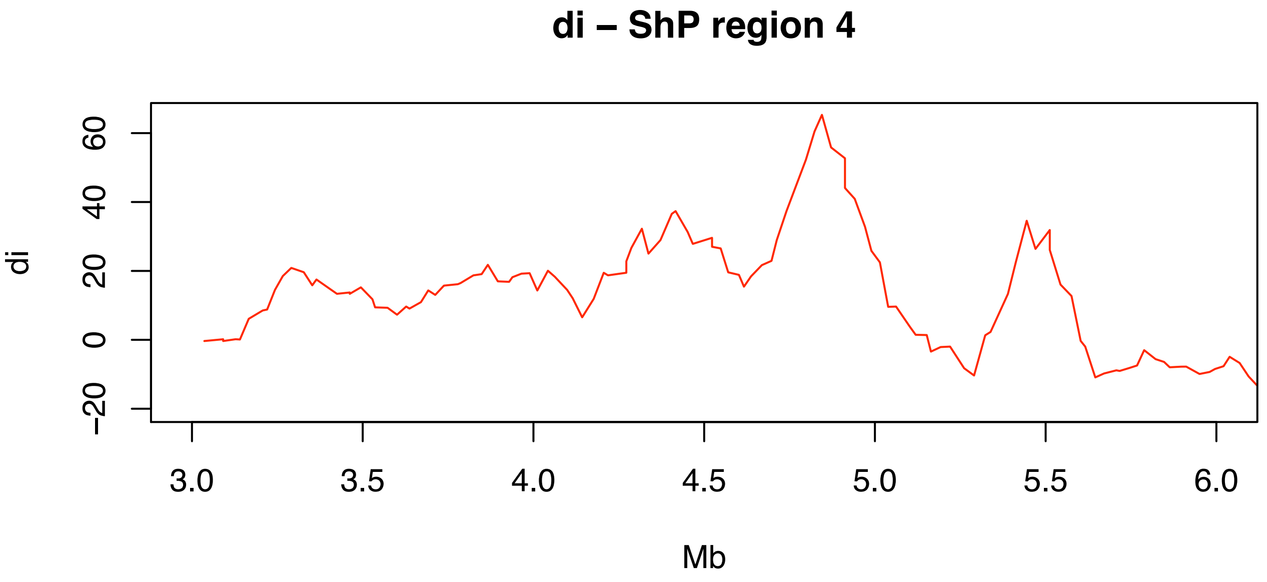
di – ShP region 4
di
60
40
20
0
−20
3.0
3.5
4.0
4.5
5.0
5.5
6.0
Mb

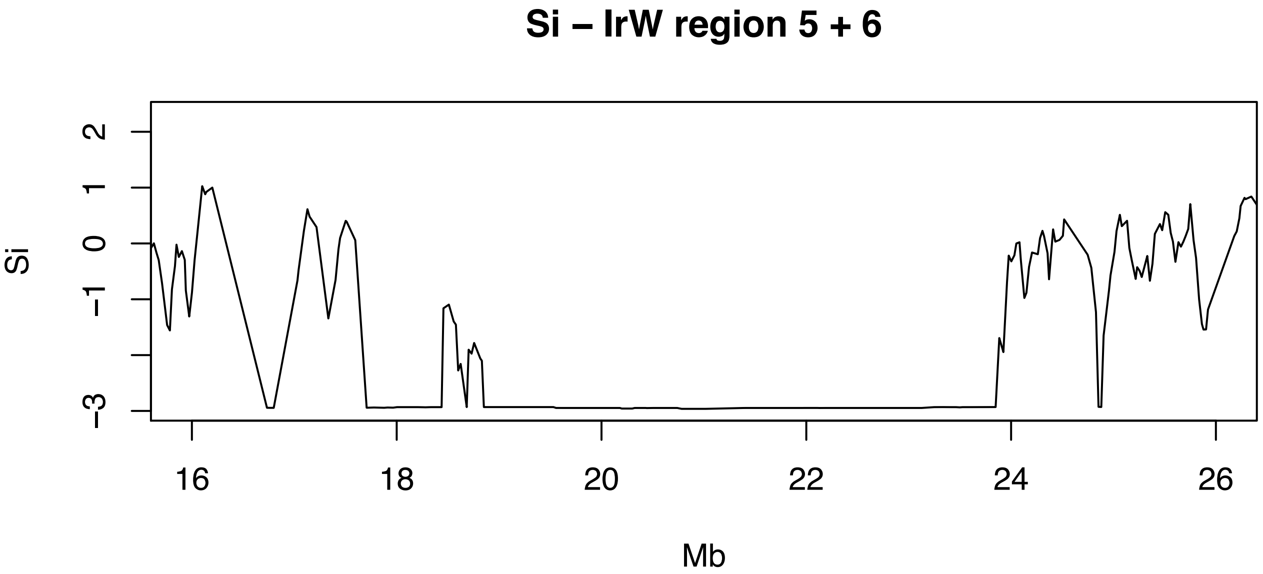
Si – lrW region 5 + 6
Si
2
1
0
−1
−3
16
18
20
22
24
26
Mb

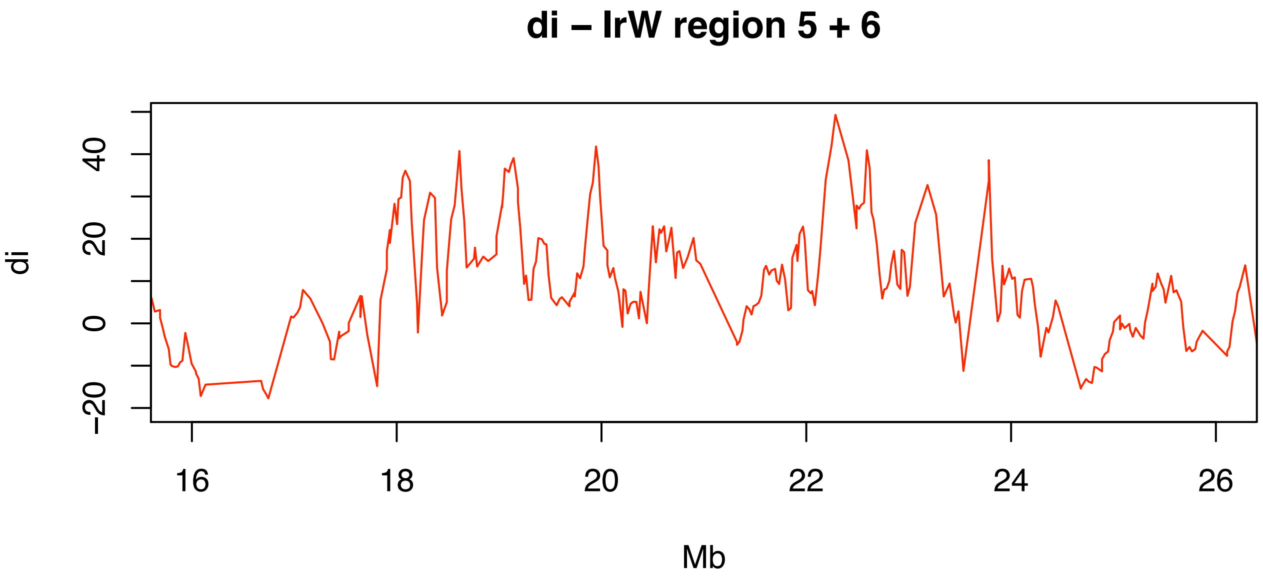
di – lrW region 5 + 6
di
40
20
0
−20
16
18
20
22
24
26
Mb

Si – Bgl region 7

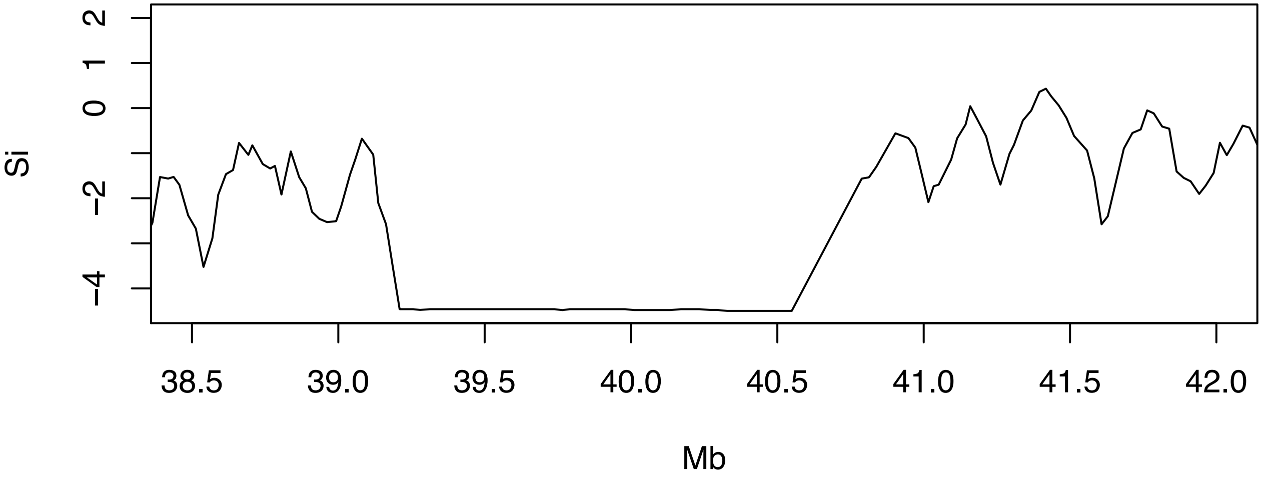

di – Bgl region 7

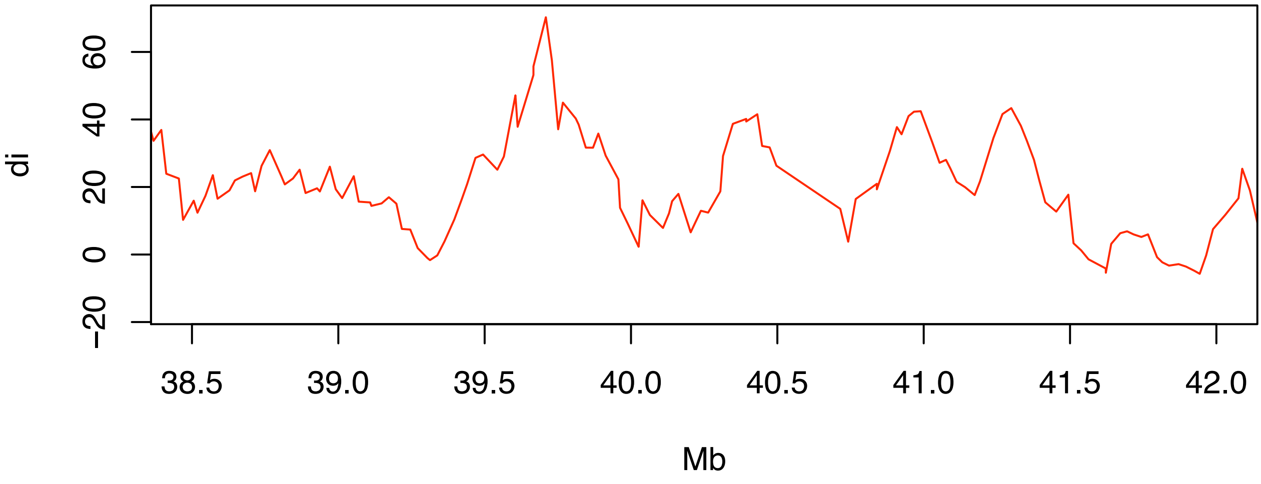

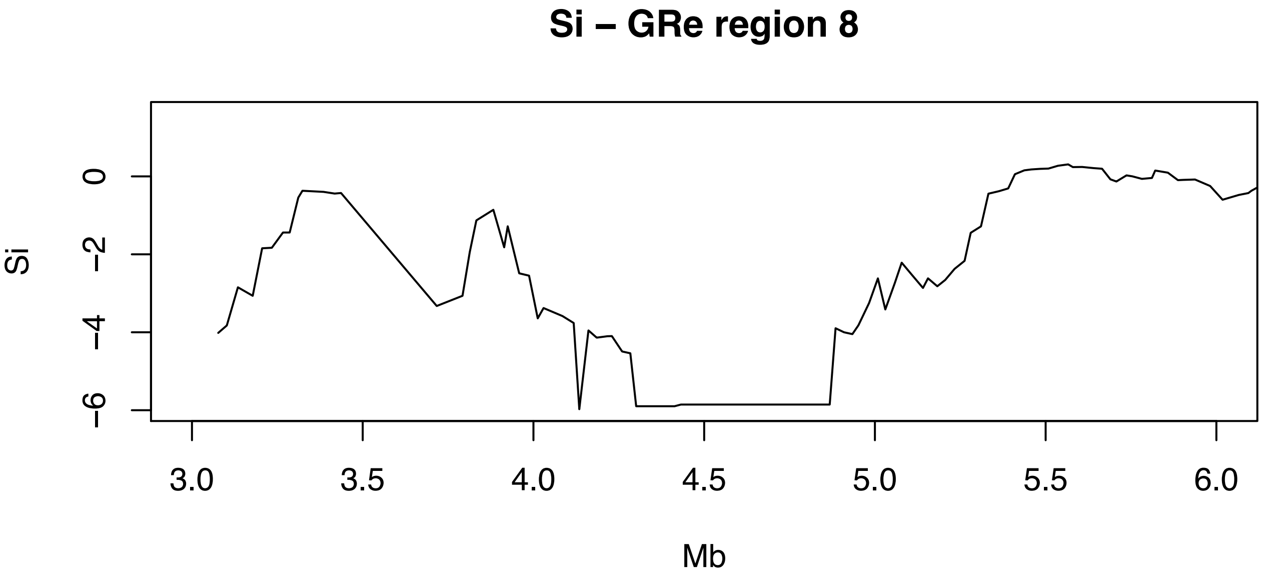

Si – GRe region 8
Si
0
−2
−4
−6
3.0
3.5
4.0
4.5
5.0
5.5
6.0
Mb

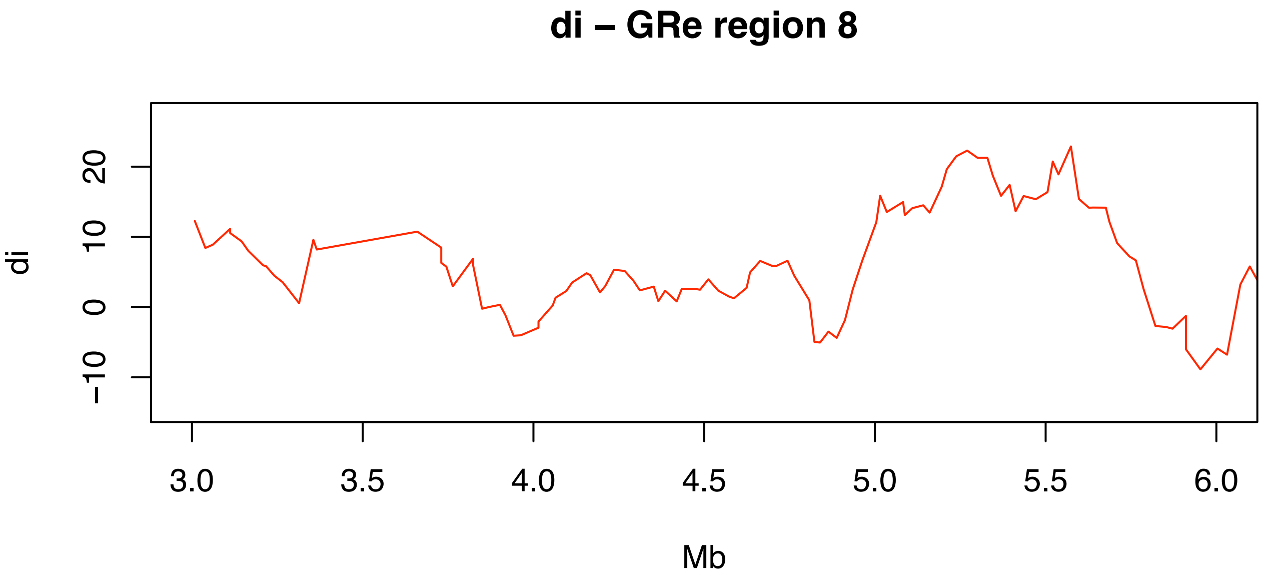

di – GRe region 8
di
20
10
0
−10
3.0
3.5
4.0
4.5
5.0
5.5
6.0
Mb

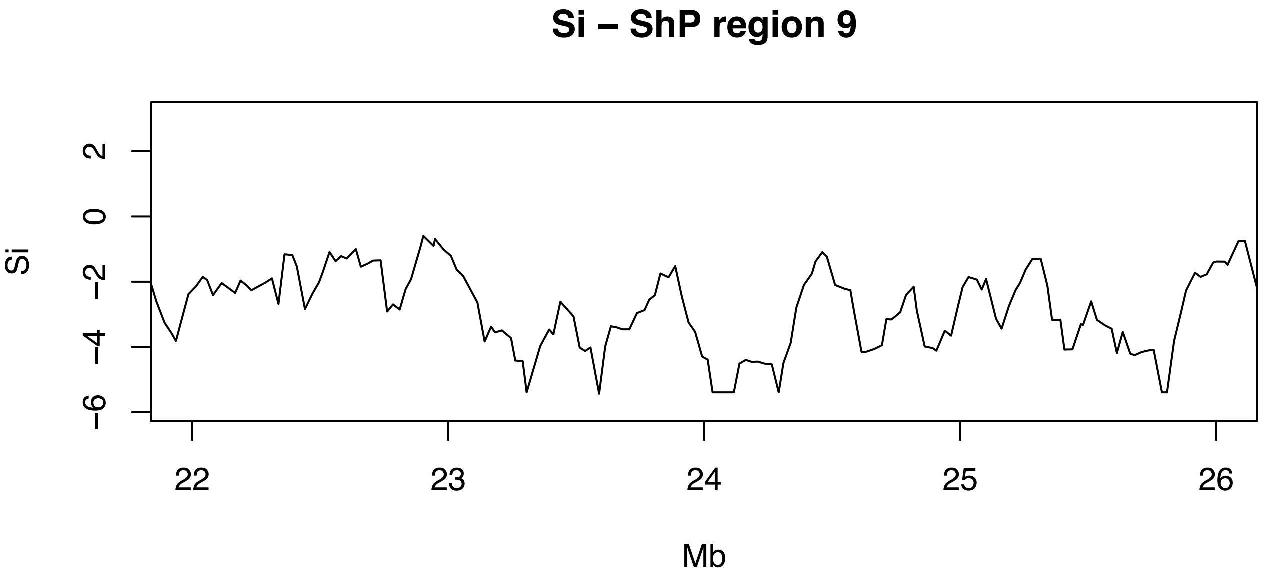

Si – ShP region 9
Si
2
0
−2
−4
−6
22
23
24
25
26
Mb

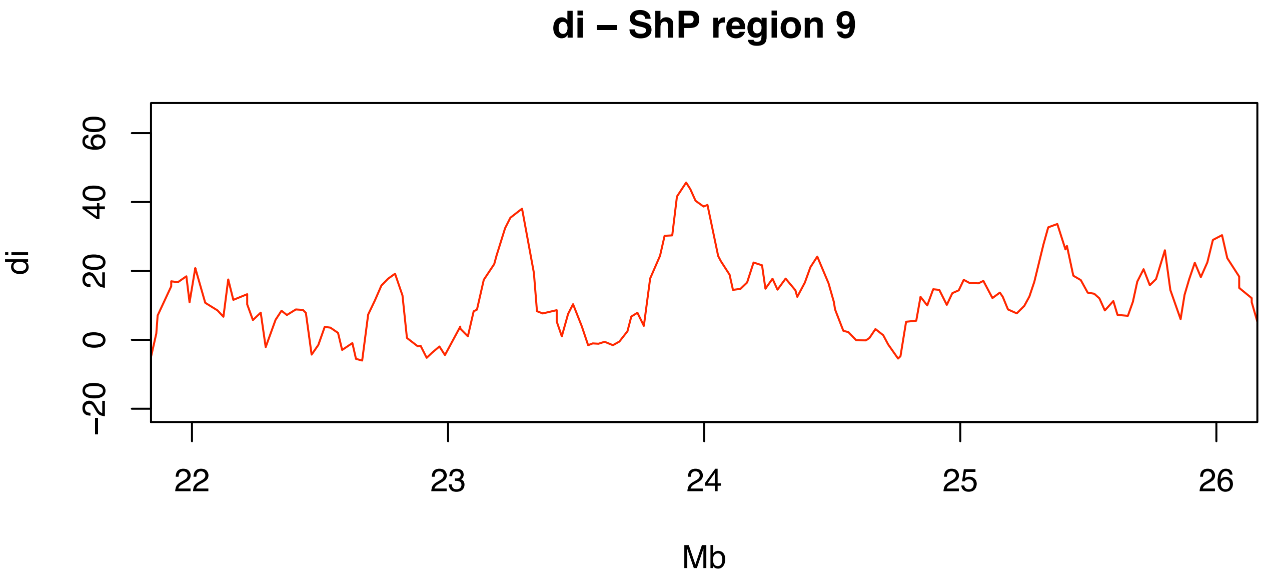

di – ShP region 9
di
60
40
20
0
−20
22
23
24
25
26
Mb

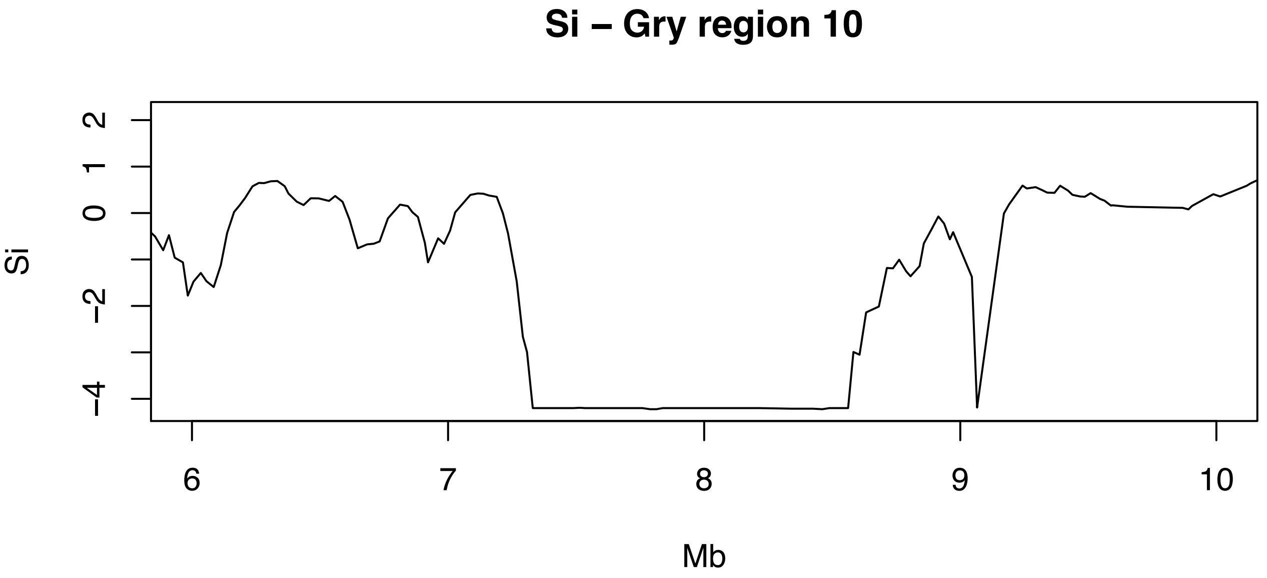
Si – Gry region 10
Si
Mb
2
1
0
−2
−4
6
7
8
9
10

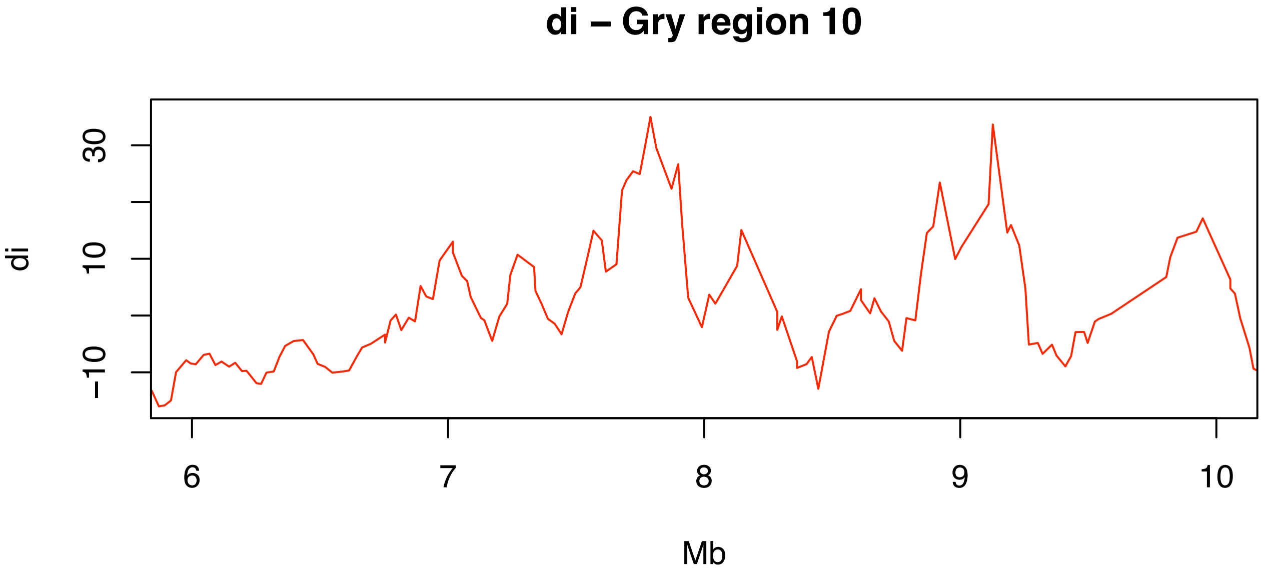
di – Gry region 10
di
Mb
30
10
−10
6
7
8
9
10
