# Supplementary material for: Identification of Genomic Regions Associated with Phenotypic Variation between Dog Breeds using Selection Mapping
Source: PLoS Genet. 2011 Oct 13;7(10):e1002316. doi: 10.1371/journal.pgen.1002316 (PMC3192833; doi:10.1371/journal.pgen.1002316)
Supplement: Table S1 — Total samples in dataset. (DOCX) [file pgen.1002316.s009.docx]

Table S1 - Total samples in dataset

| **breed** | **code** | **full dataset** | **reduced dataset** |
| --- | --- | --- | --- |
| Australian Shepherd | ASh | 1 | 0 |
| Beagle | Bgl | 10 | 10 |
| Belgian Tervuren | BeT | 12 | 12 |
| Bernese Mountain Dog | BMD | 12 | 12 |
| Border Collie | BoC | 16 | 16 |
| Border Terrier | BoT | 25 | 25 |
| Boxer | Box | 8 | 0 |
| Brittany Spaniel | BrS | 12 | 12 |
| Cavalier King Charles Spaniel | CKC | 5 | 0 |
| Chihuahua | Chi | 2 | 0 |
| Cocker Spaniel | CoS | 14 | 14 |
| Czechoslovakian Wolf Dog | CWD | 3 | 0 |
| Dachshund | Dac | 12 | 12 |
| Dalmatian | Dal | 7 | 0 |
| Doberman Pinscher | Dob | 25 | 25 |
| Elkhound | Elk | 12 | 12 |
| English Bull Terrier | EBT | 8 | 0 |
| English Bulldog | EBD | 13 | 13 |
| English Cocker Spaniel | ECS | 2 | 0 |
| English Setter | ESt | 12 | 12 |
| English Springer Spaniel | ESS | 3 | 0 |
| Eurasian | Eur | 12 | 12 |
| Finnish Spitz | FSp | 12 | 12 |
| Flatcoated Retriever | FcR | 2 | 0 |
| German Shepherd | GSh | 12 | 12 |
| Golden Retriever | GRe | 14 | 14 |
| Gordon Setter | GoS | 25 | 25 |
| Greenland Sledge Dog | GSl | 12 | 12 |
| Greyhound | Gry | 11 | 11 |
| Irish Wolfhound | IrW | 11 | 11 |
| Jack Russell Terrier | JRT | 12 | 12 |
| Labrador Retriever | LRe | 14 | 14 |
| Large Munsterlander | LMu | 1 | 0 |
| Mops | Mop | 2 | 0 |
| Newfoundland | NFd | 25 | 25 |
| Nova Scotia Duck Tolling Retriever | NSD | 23 | 23 |
| Rottweiler | Rtw | 12 | 12 |
| Samoyed | Sam | 2 | 0 |
| Sarloos | Sar | 2 | 0 |
| Schipperke | Sci | 25 | 25 |
| Schnauzer | Scn | 3 | 0 |
| Shar Pei | ShP | 11 | 11 |
| Siberian Husky | Hus | 2 | 0 |
| Standard Poodle | StP | 12 | 12 |
| Yorkshire Terrier | TYo | 12 | 12 |
| Weimaraner | Wei | 26 | 26 |
| Wolf | Wlf | 15 | 15 |
|  |  |  |  |
| **total** | **samples** | **524** | **471** |
| **total** | **breeds** | **47** | **31** |
