# Supplementary material for: Identification of Genomic Regions Associated with Phenotypic Variation between Dog Breeds using Selection Mapping
Source: PLoS Genet. 2011 Oct 13;7(10):e1002316. doi: 10.1371/journal.pgen.1002316 (PMC3192833; doi:10.1371/journal.pgen.1002316)
Supplement: Table S2 — Breed bottleneck sizes used in simulations. (DOCX) [file pgen.1002316.s010.docx]

Table S2 - Breed bottleneck sizes used in simulations. Breed bottleneck sizes (4Ne) inferred by comparing the decay of linkage disequilibrium in simulated and real data sets. Simulations were run using the same general model as described previously. In total we simulated 780 complete data sets spanning all possible combinations of breed bottle neck sizes (ranging from 0.001 - 0.03 * (ancient Wolf Ne) (each value separated by 0.0005) and sample sizes (ranging from 10-52 haplotypes). We next found the best fitting bottle neck size by comparing real and simulated LD decay using the least squares method.

| Breed | Breed bottleneck sample size |
| --- | --- |
| Belgian Tervuren | 192.1 |
| Beagle | 226 |
| Bernese Mountain Dog | 135.6 |
| Border Collie | 237.3 |
| Border Terrier | 124.3 |
| Brittany Spaniel | 372.9 |
| Cocker Spaniel | 259.9 |
| Dachshund | 463.3 |
| Doberman Pinscher | 124.3 |
| English Bulldog | 146.9 |
| Elkhound | 372.9 |
| English Setter | 237.3 |
| Eurasian | 248.6 |
| Finnish Spitz | 180.8 |
| Gordon Setter | 305.1 |
| Golden Retriever | 282.5 |
| Greyhound | 203.4 |
| German Shepherd | 158.2 |
| Greenland Sledge Dog | 226 |
| Irish Wolfhound | 113 |
| Jack Russell Terrier | 621.5 |
| Labrador Retriever | 282.5 |
| Newfoundland | 203.4 |
| Nova Scotia Duck Tolling Retriever | 158.2 |
| Rottweiler | 146.9 |
| Schipperke | 214.7 |
| Shar Pei | 497.2 |
| Standard Poodle | 237.3 |
| Terrier Yorkshire | 372.9 |
| Weimaraner | 124.3 |
