# Supplementary material for: Identification of Genomic Regions Associated with Phenotypic Variation between Dog Breeds using Selection Mapping
Source: PLoS Genet. 2011 Oct 13;7(10):e1002316. doi: 10.1371/journal.pgen.1002316 (PMC3192833; doi:10.1371/journal.pgen.1002316)
Supplement: Table S3 — Phenotypes used in across-breed GWAs. (DOCX) [file pgen.1002316.s011.docx]

Table S3 - Phenotypes used in across-breed GWAs

| Code | Boldness | Drop ear | Size (kg) | Furnish | Tail curl | Curiousity / Fear | Aggression | Sociability | Chase-proneness | Playfulness |
| --- | --- | --- | --- | --- | --- | --- | --- | --- | --- | --- |
|  | 0 (shy) | 1 (prick ear) | | 0 (absent) | 1 (straight) |  |  |  |  |  |
|  | 1 (bold) | 5 (drop ear) | | 1 (present) | 5 (tight curl) | |  |  |  |  |
|  |  |  |  |  |  |  |  |  |  |  |
| ASh | 1 | 3 | 25 | 0 |  | 3.4 | 2.1 | 3.6 | 2.5 | 3.4 |
| BeT | 1 | 1 | 32 | 0 | 2 | 3.5 | 1.9 | 3.4 | 2.8 | 3.4 |
| Bgl | 0 | 5 | 10 | 0 | 2 |  |  |  |  |  |
| BMD | 1 | 5 | 45 | 0 | 2 | 3.2 | 1.5 | 3.5 | 2.2 | 2.7 |
| BoC | 1 | 3 | 17 | 0 | 2 | 3.4 | 1.9 | 3.7 | 2.6 | 3.7 |
| BoT | 1 | 3 | 6 | 1 | 1 | 3.6 | 1.7 | 3.7 | 2.1 | 3.1 |
| Box | 1 | 2 | 29 | 0 | 1-4 | 3.7 | 1.8 | 3.9 | 2.8 | 3.4 |
| BrS | 0 | 5 | 17 | 0 |  |  |  |  |  |  |
| Chi | 0 | 1 | 2 | 0 | 2 |  |  |  |  |  |
| CKC | 0 | 5 | 6 | 0 | 2 | 3.1 | 1.8 | 3.5 | 1.9 | 2.3 |
| CoS |  | 5 |  | 0 | 2 | 3.4 | 1.7 | 3.4 | 2.2 | 2.7 |
| CWD |  | 1 |  | 0 | 2 |  |  |  |  |  |
| Dac | 0 | 5 | 7 | 0 | 1 | 3.6 | 1.8 | 3.2 | 2.1 | 2.3 |
| Dal | 1 | 5 | 25 | 0 | 1 | 3.3 | 2.1 | 3.5 | 2.7 | 3.1 |
| Dob | 1 | 2 | 35 | 0 | 1-4 | 3.5 | 1.6 | 3.3 | 2.9 | 3.6 |
| EBD | 0 | 3 | 24 | 0 |  |  |  |  |  |  |
| EBT |  | 3 |  | 0 | 1 | 4.2 | 1.7 | 3.7 | 2.0 | 3.0 |
| ECS | 0 | 5 | 14 | 0 | 2 | 3.4 | 1.7 | 3.4 | 2.2 | 2.7 |
| Elk | 1 | 1 | 23 | 0 | 5 |  |  |  |  |  |
| ESS | 0 | 5 | 23 | 0 | 2 | 3.3 | 2.0 | 3.5 | 2.2 | 3.0 |
| ESt | 0 | 5 | 31 | 0 | 2 |  |  |  |  |  |
| Eur |  | 1 |  | 0 | 4 | 3.0 | 1.7 | 3.1 | 2.0 | 1.9 |
| FcR | 0 | 4 | 29 | 0 | 2 | 3.7 | 1.9 | 3.9 | 2.7 | 3.6 |
| FSp |  | 1 |  | 0 | 5 |  |  |  |  |  |
| GoS | 0 | 5 | 31 | 0 | 2 | 3.4 | 1.6 | 3.3 | 2.5 | 2.1 |
| Gry | 0 | 3 | 30 | 0 | 1 |  |  |  |  |  |
| GRe | 0 | 4 | 32 | 0 | 2 | 3.2 | 1.7 | 3.7 | 3.0 | 3.3 |
| GSh | 1 | 1 | 37 | 0 | 2 | 3.8 | 1.8 | 3.5 | 2.7 | 3.7 |
| GSl |  | 1 |  | 0 | 4 |  |  |  |  |  |
| Hus | 1 | 1 | 24 | 0 | 3 |  |  |  |  |  |
| IrW | 0 | 3 | 54 | 1 | 2 |  |  |  |  |  |
| JRT | 1 | 3 | 7 | 0 | 1-4 | 3.4 | 1.9 | 3.4 | 2.7 | 3.4 |
| LMu |  | 5 |  | 0 | 2 |  |  |  |  |  |
| LRe | 0 | 4 | 30 | 0 | 1 | 3.9 | 1.9 | 3.8 | 3.0 | 3.5 |
| Mop |  | 3 |  | 0 | 5 |  |  |  |  |  |
| NFd | 0 | 5 | 64 | 0 | 2 | 3.4 | 1.9 | 3.7 | 2.0 | 2.8 |
| NSD | 0 | 4 | 20 | 0 | 2 | 3.1 | 1.8 | 3.5 | 2.6 | 3.4 |
| Rtw | 1 | 3 | 45 | 0 | 1-3 | 3.7 | 2.0 | 3.7 | 2.9 | 3.6 |
| Sam | 1 | 1 |  | 0 | 4 | 3.4 | 1.7 | 3.7 | 2.5 | 2.9 |
| Sar |  | 1 |  | 0 | 2 |  |  |  |  |  |
| Sci | 1 | 1 | 7 | 0 | 4 | 3.6 | 2.0 | 3.1 | 1.7 | 2.8 |
| Scn | 1 | 3 | 17 | 0 | 3 | 3.6 | 2.0 | 3.1 | 1.7 | 2.8 |
| ShP | 1 | 3 | 24 | 0 | 4 |  |  |  |  |  |
| StP | 0 | 5 | 25 | 1 | 1-5 | 3.2 | 1.9 | 3.3 | 2.2 | 3.1 |
| TYo | 1 | 2 | 3 | 1 | 3 |  |  |  |  |  |
| Wei | 0 | 5 | 28 | 0 | 2 | 3.8 | 1.9 | 3.4 | 3.3 | 3.5 |
|  |  |  |  |  |  |  |  |  |  |  |
|  |  |  |  |  |  |  |  |  |  |  |
|  |  |  |  |  |  |  |  |  |  |  |
