# Supplementary material for: Identification of Genomic Regions Associated with Phenotypic Variation between Dog Breeds using Selection Mapping
Source: PLoS Genet. 2011 Oct 13;7(10):e1002316. doi: 10.1371/journal.pgen.1002316 (PMC3192833; doi:10.1371/journal.pgen.1002316)
Supplement: Table S4 — Single-SNP FST. SNPs with minor allele frequency >0.15 and FST>0.55. Nearby SNPs <500kb are clustered into regions. (DOCX) [file pgen.1002316.s012.docx]

Table S4 - Single-SNP *F_ST_*. SNPs with minor allele frequency > 0.15 and *F_ST_* > 0.55. Nearby SNPs < 500kb are clustered into regions.

| **chrom** | **start (bp)** | **end (bp)** | **mean Fst** | **max Fst** | **no. snps** | **length (bp)** | **association** | **example genes** |
| --- | --- | --- | --- | --- | --- | --- | --- | --- |
| X | 104,640,567 | 107,235,825 | 0.67 | 0.75 | 96 | 2,595,258 | sociality, size, skull shape | many |
| 10 | 9,836,009 | 11,792,711 | 0.62 | 0.81 | 33 | 1,956,702 | drop ear, size, boldness | WIF1, HMGA2, MSRB3 |
| X | 85,365,233 | 87,444,776 | 0.57 | 0.58 | 29 | 2,079,543 | limb/tail length | many |
| 13 | 11,095,120 | 11,678,731 | 0.61 | 0.73 | 10 | 583,611 | furnishing | RSPO3 |
| 15 | 44,216,576 | 44,267,011 | 0.62 | 0.68 | 6 | 50,435 | size | IGF1 |
| 24 | 26,270,399 | 26,370,499 | 0.67 | 0.70 | 5 | 100,100 | coat colour | agouti |
| X | 27,990,332 | 28,152,042 | 0.61 | 0.63 | 5 | 161,710 |  | DMD |
| 20 | 24,841,077 | 24,889,547 | 0.58 | 0.63 | 4 | 48,470 | coat colour | MITF |
| 1 | 96,286,007 | 96,335,577 | 0.57 | 0.58 | 4 | 49,570 | snout ratio, curly tail | RCL1 |
| 25 | 3,603,872 | 4,065,978 | 0.60 | 0.63 | 3 | 462,106 |  | FOXO1, BRD2 |
| 20 | 20,449,477 | 20,539,359 | 0.58 | 0.62 | 3 | 89,882 |  | KLF15, ZXDC, UROC1, TXNRD3 |
| 13 | 10,210,459 | 10,225,305 | 0.58 | 0.59 | 3 | 14,846 |  | OXR1 |
| X | 120,769,286 | 121,212,627 | 0.70 | 0.70 | 2 | 443,341 |  | MAGEA, THEM185A |
| 31 | 14,888,449 | 14,944,938 | 0.60 | 0.61 | 2 | 56,489 |  | NRIP1 |
| 3 | 68,103,223 | 68,260,652 | 0.58 | 0.60 | 2 | 157,429 |  | CPEB2 |
| 10 | 5,221,427 | 5,440,236 | 0.58 | 0.60 | 2 | 218,809 | size | no genes |
| 3 | 93,933,450 | 93,944,095 | 0.59 | 0.60 | 2 | 10,645 | size | no genes |
| 15 | 32,638,117 | 32,853,840 | 0.56 | 0.57 | 2 | 215,723 |  | KITLG |
| 16 | 3,198,732 | 3,212,612 | 0.56 | 0.56 | 2 | 13,880 |  | PKD1L1 |
| 2 | 77,639,995 |  | 0.71 | 0.71 | 1 | 0 |  | RUNX3 |
| X | 45,730,871 |  | 0.66 | 0.66 | 1 | 0 |  | PHF8 |
| X | 31,811,399 |  | 0.65 | 0.65 | 1 | 0 |  | FTHL17 |
| 20 | 26,363,605 |  | 0.64 | 0.64 | 1 | 0 |  | FAM19A1 |
| 4 | 26,501,614 |  | 0.63 | 0.63 | 1 | 0 |  | no genes |
| 32 | 7,477,172 |  | 0.63 | 0.63 | 1 | 0 | coat type | FGF5 |
| 18 | 53,644,828 |  | 0.62 | 0.62 | 1 | 0 |  | SPTBN2 |
| X | 88,183,292 |  | 0.61 | 0.61 | 1 | 0 |  | TRPC5 |
| 32 | 8,385,378 |  | 0.61 | 0.61 | 1 | 0 |  | BMP3 |
| 26 | 11,165,766 |  | 0.60 | 0.60 | 1 | 0 |  | ATP2A2 |
| 4 | 33,687,645 |  | 0.60 | 0.60 | 1 | 0 |  | NRG3 |
| 33 | 8,929,163 |  | 0.59 | 0.59 | 1 | 0 |  | no genes |
| 27 | 5,545,082 |  | 0.58 | 0.58 | 1 | 0 | coat type | KRT71 |
| 4 | 3,242,023 |  | 0.58 | 0.58 | 1 | 0 |  | PADI4 |
| 28 | 27,342,734 |  | 0.57 | 0.57 | 1 | 0 |  | HABP2 |
| 13 | 12,260,714 |  | 0.57 | 0.57 | 1 | 0 |  | HSP90AB1 |
| 16 | 21,666,143 |  | 0.57 | 0.57 | 1 | 0 |  | SHH sonic hedgehog |
| 14 | 21,146,119 |  | 0.57 | 0.57 | 1 | 0 |  | CDK6 |
| 33 | 15,899,884 |  | 0.56 | 0.56 | 1 | 0 |  | BBX |
| 26 | 9,721,116 |  | 0.55 | 0.55 | 1 | 0 |  | DENR |
| 11 | 46,948,403 |  | 0.55 | 0.55 | 1 | 0 |  | no genes |
| 31 | 6,951,479 |  | 0.55 | 0.55 | 1 | 0 |  | SMAD2 |
| 5 | 51,639,046 |  | 0.55 | 0.55 | 1 | 0 |  | NFIA |
| 16 | 43,873,583 |  | 0.55 | 0.55 | 1 | 0 |  | PDGFRL |
| 3 | 73,085,096 |  | 0.55 | 0.55 | 1 | 0 |  | MSX1 |
