# Supplementary material for: Identification of Genomic Regions Associated with Phenotypic Variation between Dog Breeds using Selection Mapping
Source: PLoS Genet. 2011 Oct 13;7(10):e1002316. doi: 10.1371/journal.pgen.1002316 (PMC3192833; doi:10.1371/journal.pgen.1002316)
Supplement: Table S8 — (DOCX) [file pgen.1002316.s016.docx]

Table S8 - List of SNPs with evidence of sequence conservation that show complete fixation for alternate alleles in 3 drop ear and 3 prick ear breeds.

| position (bp) | reference base | drop ear allele | prick ear allele | location |
| --- | --- | --- | --- | --- |
| 9,839,420 | T | C | . | SRGAP1 intron |
| 9,883,174 | C | T | . | SRGAP1 intron |
| 10,126,827 | G | A | . | XPOT intron |
| 10,229,114 | C | . | T | RPL17 human tblastn |
| 10,646,662 | G | C | . | WIF1 exon |
| 10,647,565 | G | A | . | WIF1 intron |
| 10,650,910 | T | G | . | WIF1 intron |
| 10,851,211 | C | T | . | upstream MSRB3 |
| 11,086,490 | G | A | . | intergenic MSRB3/HMGA2 |
| 11,092,498 | G | A | . | intergenic MSRB3/HMGA2 |
| 11,096,760 | G | A | . | intergenic MSRB3/HMGA2 |
| 11,121,003 | G | A | . | intergenic MSRB3/HMGA2 |
| 11,243,713 | A | G | . | intergenic MSRB3/HMGA2 |
| 11,364,272 | T | . | C | HMGA2 intron |
| 11,364,385 | G | . | A | HMGA2 intron |
| 11,388,768 | C | . | G | HMGA2 intron |
| 11,402,964 | G | . | A | HMGA2 intron |
| 11,415,895 | G | . | T | HMGA2 intron |
| 11,421,760 | G | . | A | HMGA2 intron |
| 11,437,394 | C | . | T | HMGA2 intron |
| 11,475,954 | G | . | A | HMGA2 intron |
| 11,476,621 | G | . | A | HMGA2 intron |
| 11,566,414 | A | G | . | downstream HMGA2 |
| 11,836,464 | G | A | . | GRIP1 intron |
| 11,965,816 | A | G | . | GRIP1 intron |
