# Supplementary material for: Identification of Genomic Regions Associated with Phenotypic Variation between Dog Breeds using Selection Mapping
Source: PLoS Genet. 2011 Oct 13;7(10):e1002316. doi: 10.1371/journal.pgen.1002316 (PMC3192833; doi:10.1371/journal.pgen.1002316)
Supplement: Table S9 — (DOCX) [file pgen.1002316.s017.docx]

Table S9 - List of SNPs with evidence of sequence conservation that show complete fixation for alternate alleles in one small compared with 4 large breeds.

| position (bp) | reference base | large allele | small allele | location |
| --- | --- | --- | --- | --- |
| 10,529,500 | A | G | . | intergenic |
| 10,678,527 | T | G | . | WIF1 intron |
| 10,948,710 | C | T | . | MSRB3 intron |
| 10,977,143 | A | T | . | MSRB3 intron |
| 10,982,698 | A | G | . | MSRB3 intron |
| 11,071,179 | G | . | A | intergenic MSRB3/HMGA2 |
| 11,077,807 | G | . | C | intergenic MSRB3/HMGA2 |
| 11,086,549 | G | . | A | intergenic MSRB3/HMGA2 |
| 11,160,682 | C | . | T | intergenic MSRB3/HMGA2 |
| 11,205,040 | G | . | T | intergenic MSRB3/HMGA2 |
| 11,206,988 | A | . | G | intergenic MSRB3/HMGA2 |
| 11,244,634 | A | . | T | intergenic MSRB3/HMGA2 |
| 11,521,225 | A | . | G | downstream HMGA2 |
| 11,618,292 | C | . | G | LLPH 3'UTR |
| 11,687,367 | A | C | . | IRAK3 exon |
| 11,993,581 | A | G | . | GRIP1 intron |
| 12,251,903 | C | A | . | intergenic |
