# Supplementary material for: Identification of Genomic Regions Associated with Phenotypic Variation between Dog Breeds using Selection Mapping
Source: PLoS Genet. 2011 Oct 13;7(10):e1002316. doi: 10.1371/journal.pgen.1002316 (PMC3192833; doi:10.1371/journal.pgen.1002316)
Supplement: Table S10 — (DOCX) [file pgen.1002316.s018.docx]

**Table S10: SNP selection for the Illumina CanineHD array**

SNPs were selected from a list of 2.8 million, taken from the dog genome paper [1] and including 4353 from the resequencing of SNP-sparse regions using Illumina resequencing. Illumina design scores were obtained for all SNPs.

The genome was scanned using non-overlapping windows of length x (=11500 bp). The value of x was adjusted by trail and error to obtain a list of 203k bead types (G/C and A/T SNPs have two bead types, all others have one). Every SNP in each window was scored and ranked according to the following criteria:

| 1) Basic score = Illumina design score x 100 (-100 for those <0.3). |
| --- |
| 2) Canid phylogeny informative SNPs (heavily favoured, +1000 unless >40 repetitive flanking bases or design score <0.7). |
| 3) Presence on previous Affymetrix arrays (heavily favoured, +1000, if not >50 repetitive flanking bases or design score <0.5) |
| 4) Presence on previous Illumina arrays (heavily favoured, +1000, if not >50 repetitive flanking bases or design score <0.5) |
| 5) Only identified as a difference between dog and other canid (penalized, -100) |
| 6) Only identified as a difference between dog and wolf (penalized, -40) |
| 7) More than 2 alleles seen (penalized, -50) |
| 8) Only identified in boxer-poodle comparison (slightly penalized, -10) |
| 9) SNPs with 2 beadtypes were penalized (-7) |
| 9) Penalty for the amount of repetitive sequence in the 120 bp flanking region (-2 x number of repetitive bases) |
| 10) SNPs close to the beginning of each window were slightly favoured, in order to make a more even coverage (-10*proportion of distance to end of window). |

All SNPs with scores over 100 were kept. This means that all SNPs on the previous Affymetrix or Illumina arrays (3,4), or in the canid list (2) are kept unless they have very low design scores and/or a lot of repetitive flanking sequence. If there were no previous Affymetrix or Illumina SNPs in each window, then the highest scoring SNP was chosen.

The list of 203k bead types was submitted to Illumina, along with 13 Y specific SNPs identified from ref. [56]. Potential duplicates or incompatibilities between primers were identified. These problematic SNPs were removed, and the final SNP list was edited manually to produce a list of 200k bead types. This was done by removing SNPs with the smallest distance to other SNPs, as long as they were not in one of the classes from 2,3 or 4.
